# Spatial, seasonal and climatic predictive models of Rift Valley fever disease across Africa

**DOI:** 10.1098/rstb.2016.0165

**Published:** 2017-06-05

**Authors:** David W. Redding, Sonia Tiedt, Gianni Lo Iacono, Bernard Bett, Kate E. Jones

**Affiliations:** 1 Centre for Biodiversity and Environment Research, Department of Genetics, Evolution and Environment, University College London, Gower Street, London WC1E 6BT, UK; 2 Department of Veterinary Medicine, Disease Dynamics Unit, University of Cambridge, Madingley Road, Cambridge CB3 0ES, UK; 3 Environmental Change, Public Health England, Didcot OX11 0RQ, UK; 4 International Livestock Research Institute, PO Box 30709-00100, Nairobi, Kenya; 5 Institute of Zoology, Zoological Society of London, Regent's Park, London NW1 4RY, UK

**Keywords:** Africa, Bayesian spatial model, climatic oscillations, Integrated Laplace Approximations, Rift Valley fever, risk map

## Abstract

Understanding the emergence and subsequent spread of human infectious diseases is a critical global challenge, especially for high-impact zoonotic and vector-borne diseases. Global climate and land-use change are likely to alter host and vector distributions, but understanding the impact of these changes on the burden of infectious diseases is difficult. Here, we use a Bayesian spatial model to investigate environmental drivers of one of the most important diseases in Africa, Rift Valley fever (RVF). The model uses a hierarchical approach to determine how environmental drivers vary both spatially and seasonally, and incorporates the effects of key climatic oscillations, to produce a continental risk map of RVF in livestock (as a proxy for human RVF risk). We find RVF risk has a distinct seasonal spatial pattern influenced by climatic variation, with the majority of cases occurring in South Africa and Kenya in the first half of an El Niño year. Irrigation, rainfall and human population density were the main drivers of RVF cases, independent of seasonal, climatic or spatial variation. By accounting more subtly for the patterns in RVF data, we better determine the importance of underlying environmental drivers, and also make space- and time-sensitive predictions to better direct future surveillance resources.

This article is part of the themed issue ‘One Health for a changing world: zoonoses, ecosystems and human well-being’.

## 1. Introduction

Emerging infectious diseases (EIDs) are a significant threat to global economies and human health [1]. They pose particularly severe healthcare challenges for resource-limited countries, and frequently place substantial economic burdens on the most vulnerable [2]. Understanding the emergence and subsequent spread of EIDs is a critical global challenge, especially for high-impact zoonotic (diseases with an animal origin) and vector-borne diseases (e.g. Ebola, Zika). Although global climate and land-use change are likely to alter reservoir host and vector distributions, understanding the impact of these changes on future disease risk is challenging [3]. This is largely because of a lack of a detailed mechanistic understanding of how reservoir host or vectors respond to environmental changes for many EIDs, or how humans and host or vector populations subsequently come into contact within a changing environment [3,4].

One approach to understanding disease risk across environments is to use non-mechanistic correlative methods, e.g. landscape epidemiology [5]. In these studies, correlative patterns between disease cases and a suite of likely environmental covariates are modelled, using methods such as MaxEnt [6] or Boosted Regression Trees (BRT) [7]. This approach can give insight into both the underlying causal environmental drivers of the disease and, by using covariate-based interpolation, the likely risk across the landscape (e.g. [8–11]). However, these types of correlative approaches ignore the underlying mechanisms of disease emergence and transmission, and may be impacted by data sampling bias, sparse and patchily distributed data—situations that are common with disease case data [3,12]. Building complexity into a correlative approach has recently become more tractable using methods such as Bayesian hierarchical spatial models solved using Integrated Laplace Approximations (INLA) [13]. This approach uses a flexible additive modelling structure to explicitly incorporate a wide variety of fixed and random effects, such as spatial and temporal autocorrelation. Currently, there have been few studies that include complex hierarchical spatial models in correlative infectious disease analyses (e.g. [14]), partially because of the previous difficulties of implementation.

A Bayesian hierarchical spatial modelling approach therefore is likely to be particularly suited for EIDs due to patchy data coverage and complex emergence and transmission patterns. One such disease, Rift Valley fever (RVF) has become one of the most important zoonoses of sub-Saharan Africa over the last century, causing devastating health and economic impacts on domestic ruminants and humans [15], and more recently causing serious epizootics outside Africa (Saudi Arabia and Yemen [16]). RVF is also a potential threat for Europe and the USA [17]. Most RVF epidemics are believed to be triggered by the emergence of unusually large numbers of adult mosquitoes transmitting RVF virus (RVFV, Family Bunyaviridae), especially of the genus *Aedes* and *Culex* [18]. Mosquito distribution and emergence, in turn, is strongly linked to ecological and climatic conditions, such as heavy rainfall and flooding [19], and to human activities that increase standing water, such as irrigation and dam building [16]. Social activities, such as human and animal gathering during the Eid al Adha religious feast, also appear to contribute to the transmission and dispersal of the disease [20]. How RVFV is maintained during inter-epidemic periods is less clear. The most commonly accepted theory is that RVFV can be maintained over several years by vertical transmission in floodwater *Aedes* mosquitoes during dry periods, and at animal watering sites by horizontal transmission between livestock and mosquitoes during rainy periods [21]. Additionally, high levels of RVF seropositivity have been noted in buffalo populations (*Syncerus caffer*), and it has been suggested that RVFV could circulate long term in wild animals, providing a persistent source of reinfection of cattle populations [21,22].

A number of studies have developed RVF monitoring and risk mapping with correlative approaches using a variety of environmental measurements, including surface temperatures, rainfall, soil type and vegetation density (NDVI) [23–25], with a strong emphasis on capturing the environmental conditions that suit the vectors. Such approaches have been used successfully to forecast outbreaks and have proven useful in the allocation of surveillance efforts [23,26]. However, these studies have tended to focus on particular African regions (e.g. Kenya and South Africa) and understanding the risk of RVF across other areas is confounded by lack of dedicated resources and under-reporting [27,28]. Fitting a single model across the entire endemic region to capture RVF's complex disease transmission processes is problematic [29] and spatially invariant models are unlikely to capture the range of environmental–disease interactions for such a geographically widespread disease. For instance, key drivers in temperate regions are likely to differ from those that are important in tropical monsoon climates. Furthermore, when testing spatially invariant models under different climatic oscillation scenarios (e.g. El Niño-Southern Oscillation, ENSO), the impacts are unlikely to be spatially and temporally heterogeneous. For instance, strong El Niño events cause high rainfall in the Horn of Africa while simultaneously generating lower than average rainfall in Malawi and South Africa [30].

Here, we improve on previous attempts and construct a spatial and temporal risk map for continental Africa by employing a flexible Bayesian hierarchical modelling approach (using INLA), to better understand the different drivers of spatial risk in RVF outbreaks. By accounting for spatial and temporal heterogeneity, we determine the importance of underlying drivers, and also make space- and time-sensitive predictions to better direct surveillance resources at the continental level.

## 2. Material and methods

### (a) Rift Valley fever occurrence data

Owing to a lack of data, we modelled the risk of RVF to livestock rather than the risk directly to humans. A major source of infection for humans is through slaughtering infected livestock [15], and high-risk conditions for animals will confer a higher risk to people. Additionally, we are, in most part, modelling the environmental conditions suitable for the disease-carrying vectors. Therefore, if there are infected vectors present, then the RVF risk to farm workers and other local people is likely intensified. RVF livestock occurrence records were collated from the Global Animal Disease Information System (EMPRES-i; http://empres-i.fao.org) from 2004 to 2016 (electronic supplementary material, table S1). We collated the following information from each RVF occurrence contained in the database: latitude and longitude (DEC), the observation date, the reporting date (observation date was used in preference to reporting date) and the predicted number of animals at risk at the locality. The coordinates for each record represented the exact location for small outbreaks, the centroid of the districts/counties affected for large outbreaks or, for outbreaks where exact location is not known, the centroid of general area of occurrence. For non-exact coordinates, environmental covariates extracted at the estimated location may not represent the value at the unknown true location. However, this is unlikely to be a problem at this analysis scale, as the covariates in our models are relatively invariant over small areas. Observations were reported from Botswana, Kenya, Madagascar, Mauritania, Namibia, Senegal, South Africa, Sudan and Swaziland, with by far the majority (90%) coming from South Africa (electronic supplementary material, table S1). EMPRES-i records that were clearly duplicates, or recorded as ‘Denied’, were removed, and records were then manually checked for incorrect coordinates by verifying that the given location matched the country recorded in the ‘Country’ column of the dataset. The final dataset contained 976 outbreak records.

### (b) Spatial environmental and habitat data

We collated spatial environmental and habitat variables for continental Africa (electronic supplementary material, table S2), and used those spatial variables linked to RVF outbreaks, or those that are thought to explain higher incidence or severity. For instance, the development and survival rates of mosquito vectors are known to be highly dependent on temperature, and as mosquitoes require water bodies for larval development, rainfall, soil type, presence of irrigation and previous history of flooding can all be employed to capture the likelihood of a grid cell containing standing water [31]. All of these variables represented synoptic data and, therefore, outbreaks occurring at the same locations had the same values irrespective of date of outbreak. We chose this approach as using data corresponding to the date of the outbreak would only have been possible for a few of the variables, reducing the available covariates. Furthermore, it is not clear over what time frame to summarize the input variables, as there may be complex interactions at play, such as flooding being caused by either consistent rain in a clay soil area or sudden rain in more porous location. Out of the initial set of variables, we retained 24 of the most orthogonal (less than 75% correlation), to give a final dataset consisting of: (i) bioclimatic variables from Hijmans *et al*. [32]: (a) BioClim BIO1 annual mean temperature; (b) BioClim BIO5 maximum temperature of warmest month; (c) BioClim BIO6 minimum temperature of coldest month; (d) BioClim BIO7 temperature annual range; (e) BioClim BIO12 annual precipitation; (f) BioClim BIO13 precipitation of wettest month; (g) BioClim BIO14 precipitation of driest month; and (h) altitude (m a.s.l.); (ii) rain event variables from Dartmouth Flood Observatory [33]: number of extreme rain events and number of major floods from 1998 to 2009; (iii) gridded livestock of the world variables from Robinson *et al*. [34]: sheep and cattle density calculated as the number of sheep or cattle per grid cell; (iv) proportion of cultivated land from harmonized land use [35]; (v) presence of irrigation from HarvestChoice [36]; (vi) human population density (2010) from the *Gridded population of the world v. 3* [37]; (vii) percentage land cover from MODIS [38], where we aggregated 16 MODIS land-cover categories into seven broad habitat classes: forest (MODIS categories 1–5), shrubland (MODIS 6–7), savannah (MODIS 8–9), grassland (MODIS 10), wetland (MODIS 11), anthropogenic (MODIS 12–14) and bare (MODIS 15–16); (viii) soil and vegetation variables from the World Soil Database [39]: % clay, organic carbon, silt, and gravel, soil pH and Leaf Area Index; (ix) total number of animals at risk of RVF (collected at each record locality to account for sample size; EMPRES-i); and (x) probability of occurrence of buffalo (*S. caffer*) calculated by a species distribution model from Tiedt [40], using data from the Global Biodiversity Information Facility [41] and calculated with a BRT model [7]. For analysis, all variables were reduced in latitudinal extent to 37° N 40.5° S and longitudinal extent to 54° E 18° W and resampled to a 0.0416° grid cell size using a World Geodetic System 84 projection using ‘raster’ [42].

### (c) Statistical analyses

The RVF data were likely to contain spatial biases as the majority of cases were reported from South Africa (electronic supplementary material, table S1) and none from known infected countries (e.g. Zambia, Zimbabwe). To reduce the impact of spatial biases on the slope estimates on the covariates, we used a Stochastic Partial Differential Equations approach [43] to fit a Gaussian random field to account for the effects of sampling different locations with different intensities. We included this random field as a spatially structured random effect using a Matérn covariance function, in a Bayesian additive regression model. We tested several different versions of the spatial mesh, choosing the appropriate complexity using Watanabe-AIC (wAIC) [44] to estimate the optimal complexity of the spatial term versus the time taken to compute the model. To sample the widest range of possible environmental conditions with minimum number of points, we sampled approximately every four latitudinal or longitudinal degrees across Africa and used these 1040 points as pseudo-absence points, with a year and month randomly assigned from uniform distributions (runif, [45]). We used a presence-background model because simulations using input data with a similar degree of clumping as our input dataset show that this type of model performs well [46]. Modelling using Gaussian point processes offers an exciting alternative method [47] and can also be inferred using an INLA Bayesian approach, but these have yet to be evaluated against presence-background methods using very clumped input data. We repeated the analysis with approximately 5° and 3° grids and with different year and month designations, but as this made no qualitative differences to the results, we use the 4° grid hereafter.

Model inference was undertaken using INLA in R (R-INLA, [13]). INLA was chosen as it represents an analytical short cut to estimate Bayesian regression parameters [13], without the need to employ, for instance, computationally expensive Markov Chain Monte Carlo algorithms [48]. INLA also has been shown to perform well with potentially very clumped and biased data compared with other common inference methods such as BRT or MaxEnt [46]. We predicted the presence/absence of RVF cases using all 24 of the most orthogonal environmental and habitat covariates, fitting both linear and square terms, so that nonlinear relationships between the dependent and independent variables were available for selection. Simple terms rather than, for instance, Bayesian splines, were chosen due to computational cost and also so that the resulting slope estimates would be easy to interpret. We used a binomial ‘error family’ for the dependent variable (presence/absence RVF). We evaluated the fit of the model using a three-pronged approach. First, we used the conditional predictive ordinate (CPO) measure of fit, which gives the probability of each individual data point given the model. CPO ranges from 0 in the case of poor fit, to *N* (the sample size) in the case of perfect fit for each data point [49]. Second, the probability integral transform, a diagnostic method used to assess whether a data variable comes from a specified distribution, in this case binomial (analogous to *Q*–*Q* plot comparison), which has an ideal result of uniform distribution of values across the range of dependent variable [49].

RVF cases were modelled within four periods across the year: quarter 1 (January–March), quarter 2 (April–June), quarter 3 (July–September) and quarter 4 (October–December), with these periods selected to capture discrete differences in rainfall and temperature throughout Africa. Cases were also modelled within three climatic oscillation groups determined by the Oceanic Niño Index (ONI; http://www.cpc.ncep.noaa.gov/products/analysis_monitoring/ensostuff/ensoyears.shtml): La Niña—cases occurring during months with an ONI score less than −1; no event—cases occurring during months with an ONI score between −1 and 1; and El Niño—cases occurring during months with an ONI score greater than 1. There were four major ENSO oscillations from 2004 to 2016. We note that some outbreaks were reported well after they were thought to have occurred, so these may have some recall bias, though this was not explicitly modelled here due to computational constraints. We then created a nested structure of 12 possible states by combining these four seasonal and three climatic oscillation groupings (table 1). These 12 groups were modelled simultaneously using an ‘iid’ term for both the intercept and slope parameters [50] which allowed us to specify a group-specific slope and intercept parameter using exchangeable hyper-priors, specified with a log-gamma distribution. We used a forward stepwise procedure to select a minimum model to aid interpretation of the remaining variables, using bespoke code (https://github.com/timcdlucas/INLAutils/blob/master/R/stepINLA.R). The univariate model with the lowest wAIC value was used as the starting model, with each ‘next best’ predictor added in turn. We used an information-theoretic approach to compare models using wAIC [44]. This algorithm avoids the model over-fitting by penalizing terms that explain little variance [51]. Using this approach, we made full Bayesian predictions at 4160 points in a 1° grid across Africa for 12 different seasonal and climatological scenarios.

**Table 1.** Number of RVF occurrence records from 2004 to 2016 split by country, seasonally and by climatic oscillation type. Quarters represent four seasonal periods: quarter 1, January–March; quarter 2, April–June; quarter 3, July–September; and quarter 4, October–December. ENSO groups were based on the Oceanic Ninõ Index: LA, La Niña (cases occurring during months with an ONI score less than −1); NE, no event (cases occurring during months with an ONI score between −1 and 1); and EN, El Niño (cases occurring during months with an ONI score greater than 1). RVF livestock occurrence records (*n* = 976) were collated from RVF data from the Global Animal Disease Information System (EMPRES-i; http://empres-i.fao.org) (electronic supplementary material, table S1).

	season and ENSO event type											
	quarter 1			quarter 2			quarter 3			quarter 4		
country	LA	NE	EN	LA	NE	EN	LA	NE	EN	LA	NE	EN
Algeria	0	0	0	1	0	0	0	0	0	0	0	0
Botswana	0	0	0	0	0	1	0	2	0	0	0	0
Chad	0	1	0	0	0	0	0	0	0	0	0	0
Comoros	0	0	0	0	0	0	0	3	0	0	0	0
Egypt	1	0	0	0	0	0	0	0	0	0	0	0
Kenya	0	0	27	0	1	0	0	3	0	0	0	31
Madagascar	4	0	1	8	1	0	1	5	0	0	12	0
Mali	0	0	0	0	0	0	1	3	0	0	0	0
Mauritania	0	0	0	0	0	0	0	35	1	3	11	4
Mayotte	0	0	0	0	0	0	1	0	0	0	0	0
Mozambique	6	0	0	0	0	0	4	0	0	0	0	0
Namibia	0	0	0	2	5	8	0	0	0	0	0	0
Senegal	0	1	1	0	0	0	0	14	0	0	6	6
Somalia	0	0	1	0	0	0	0	0	0	0	0	0
South Africa	100	0	314	73	13	169	0	4	0	1	18	1
South Sudan	0	0	0	0	0	0	0	0	0	0	1	0
Sudan	0	0	1	0	0	0	0	0	0	1	2	0
Swaziland	0	0	0	1	0	0	0	1	0	0	0	0
Uganda	0	26	4	0	0	0	0	0	0	0	0	0
Tanzania	0	0	24	1	6	0	0	0	0	0	0	1
Western Sahara	0	0	0	6	0	0	0	0	0	0	0	0

## 3. Results

### (a) Seasonal and climatic spatial patterns of Rift Valley fever cases

We find that RVF risk has distinct seasonal and climatic spatial patterns. Across the four seasonal periods, there were marked differences in the numbers of RVF cases recorded, where quarters 1 and 2 (January–June) contained the majority of all cases (82%; table 1). Numbers of RVF cases were higher overall in years with either strong El Niño or La Niña oscillations. The majority of cases occurring in strong El Niño event years were located in South Africa or Kenya. In comparison, in strong La Niña event years, while RVF cases were again predominately in South Africa (173 of 215), they were also recorded in North African countries (Western Sahara, Algeria, Egypt) in smaller numbers (eight recorded cases; table 1).

### (b) Drivers of Rift Valley fever risk

We find that RVF risk increased with the presence of irrigation, a larger proportion of land under cultivation and a higher human population density (INLA regression model, *n* = 976, wAIC = 776.18). The mean annual rainfall had a negative impact on RVF risk (table 2; electronic supplementary material, figure S1). Our cross-validation showed the INLA model fitted with a right-skewed CPO histogram (electronic supplementary material, figure S2), indicating that most of the values had high probability when systematically removed from the data [47]. Models that contained the spatial autocorrelation always had a lower wAIC score than those without (wAIC = 1091.75–981.13), validating the inclusion of a spatial term.

**Table 2.** Slope values from a minimum Bayesian additive regression model for RVF occurrence records from 2004 to 2016 independent of spatial, seasonal and climatic oscillation effects estimated using INLA. Percentage land-use classes are aggregated from 16 MODIS [38] habitat classes (see Material and methods). Mean and s.d. represent the mean and standard deviation of the slope values; 0.025, 0.5, 0.975 represent quantiles of the distribution (see electronic supplementary material, figure S1 for slope distributions). Italics denotes variable had a slope mean value that was significantly greater than zero.

variable	mean	s.d.	0.025	0.5	0.975
intercept	−0.0865	11.182	−22.0406	−0.0868	21.8493
ann. precipitation	0.1571	0.09	−0.0202	0.1573	0.3334
ann. precipitation^2^	−0.0206	0.0089	*−0.0381*	*−0.0206*	*−0.0031*
ann. mean temp.	0.0021	0.0045	−0.0067	0.0021	0.011
ann. mean temp.^2^	0	0	0	0	0
no. of observed floods	0.0059	0.0093	−0.0126	0.0059	0.0243
sheep density	0.0146	0.0206	−0.0258	0.0146	0.0548
cattle density	−0.0033	0.0125	−0.0283	−0.0031	0.0209
prop. of cultivated land	−0.439	0.2335	−0.8975	−0.439	0.019
prop. of cultivated land^2^	1.6028	0.6198	*0.3854*	*1.6029*	*2.8183*
presence of irrigation	0.0473	0.0076	*0.0328*	*0.0472*	*0.0627*
human popn density	0.0169	0.0079	*0.0015*	*0.0169*	*0.0326*
% forest	0.0059	11.1761	−21.9365	0.0056	21.93
% shrubland	−0.0465	11.176	−21.9888	−0.0469	21.8774
% savannah	0.0199	11.176	−21.9223	0.0196	21.9438
% grassland	0.0576	11.176	−21.8846	0.0573	21.9815
% wetland	−0.1453	11.1769	−22.0894	−0.1456	21.7804
% anthro.	0.0654	11.176	−21.8768	0.0651	21.9893
% bare	−0.0467	11.176	−21.989	−0.047	21.8773
no. of animals at risk	−0.0197	0.0148	−0.0531	−0.0176	0.0056

### (c) Spatial predictions of Rift Valley fever risk

**Figure 1.**
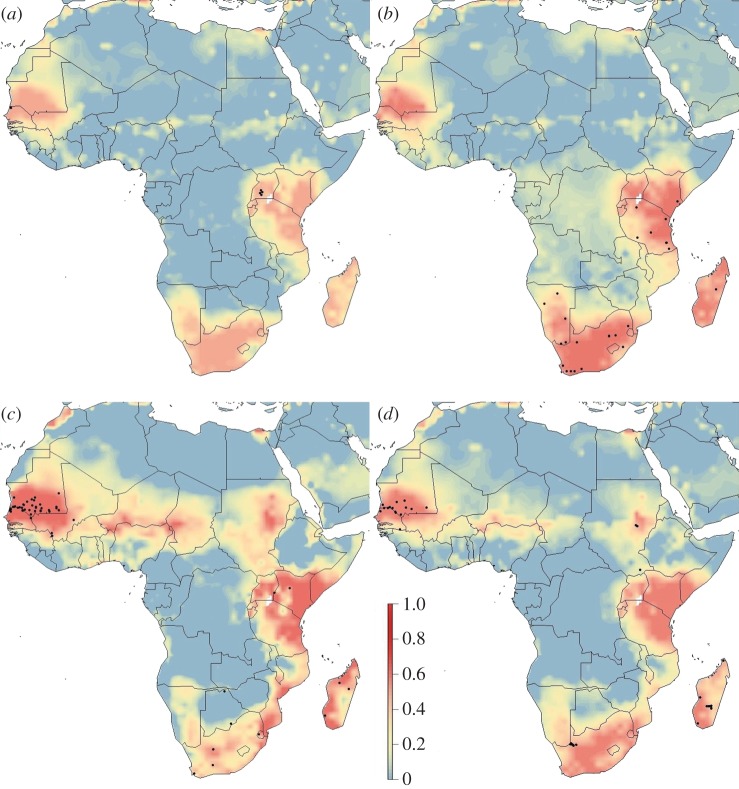
Spatial distribution of RVF risk for years without an ENSO event for (*a*) quarter 1, January–March; (*b*) quarter 2, April–June; (*c*) quarter 3, July–September; and (*d*) quarter 4, October–December. Risk was estimated from occurrence records from 2004 to 2016 with a Bayesian additive regression model using INLA. Risk probability per 1° grid cell is represented on a linear colour scale from 1 to 0, where red is most suitable given the environmental conditions and dark blue unsuitable. Axis labels indicate degrees, in a World Geodetic System 84 projection. Filled black circles represent locations of historic RVF outbreaks (see electronic supplementary material, table S1).

**Figure 2.**
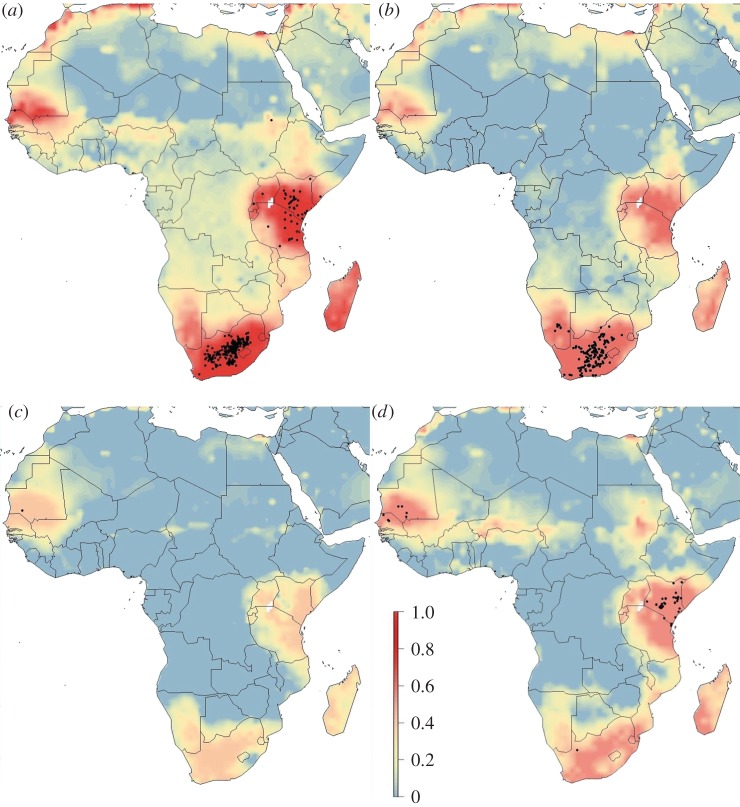
Spatial distribution of RVF risk for years with a strong El Niño event for (*a*) quarter 1, January–March; (*b*) quarter 2, April–June; (*c*) quarter 3, July–September; and (*d*) quarter 4, October–December. Risk was estimated from occurrence records from 2004 to 2016 with a Bayesian additive regression model using INLA. Risk probability per 1° grid cell is represented on a linear colour scale from 1 to 0, where red is most suitable given the environmental conditions and dark blue unsuitable. Axis labels indicate degrees, in a World Geodetic System 84 projection. Filled black circles represent locations of historic RVF outbreaks (see electronic supplementary material, table S1).

**Figure 3.**
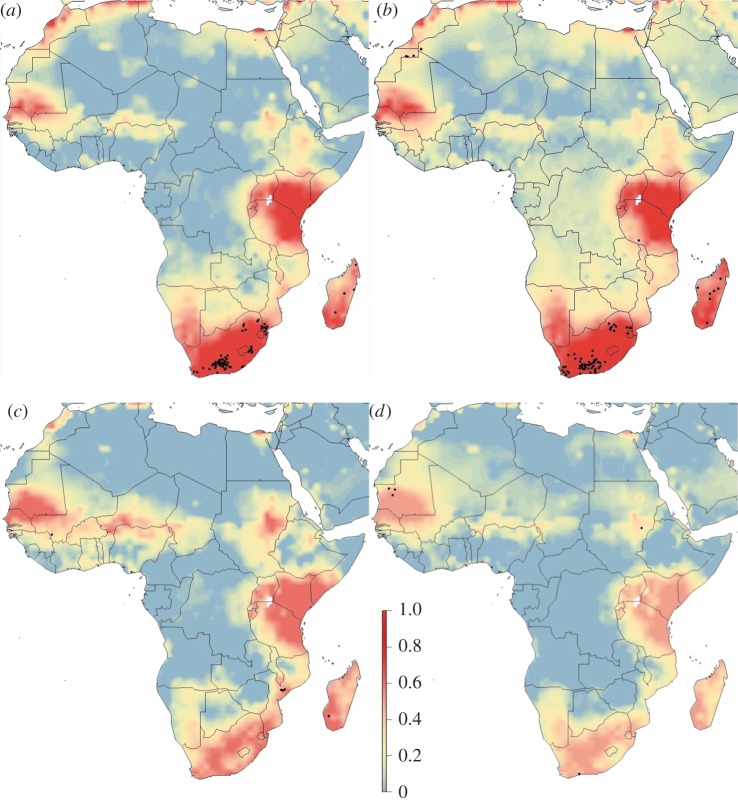
Spatial distribution of RVF risk for years with a strong La Niña event for (*a*) quarter 1, January–March; (*b*) quarter 2, April–June; (*c*) quarter 3, July–September; and (*d*) quarter 4, October–December. Risk was estimated from occurrence records from 2004 to 2016 with a Bayesian additive regression model using INLA. Risk probability per 1° grid cell is represented on a linear colour scale from 1 to 0, where red is most suitable given the environmental conditions and dark blue unsuitable. Axis labels indicate degrees, in a World Geodetic System 84 projection. Filled black circles represent locations of historic RVF outbreaks (see electronic supplementary material, table S1).

Spatial predictions of RVF risk using the best model varied seasonally and within climatic oscillations (figures 1–3). Years with no designated climatic oscillations show patterns of low risk in quarters 1 and 2 (January–June), but high and widespread risk in western Africa (distinct peaks in Chad and Niger), though these peaks occur in areas with likely lower predictive confidence (electronic supplementary material, figure S3), and southern areas of Sudan in quarters 3 and 4 (July–December), and high risk in both South Africa and Madagascar in quarter 4 (figure 1). By contrast, for both strong El Niño and La Niña event years, quarters 3 and 4 are predicted low risk, while quarters 1 and 2 have many high-risk areas, including the endemic areas (figures 2 and 3). For strong El Niño event years, quarter 1 had especially high-risk areas, as expected, especially in South Africa, and in Kenya–Tanzania–Uganda due to the high numbers of recorded cases there (figure 2). For both strong El Niño and La Niña event years, there were similar spatial patterns of RVF risk except for Madagascar and western Africa which had higher expected risk in La Niña event years, and the Chad–Niger–Sudan risk peaks which were higher in quarter 3 in La Niña event years (figure 3).

## 4. Discussion

Irrespective of the location where an outbreak occurred or the time of year that it happened, the presence of agricultural irrigation in the local area remains strongly linked to an increased risk of RVF. This is understandable as irrigation is known to directly benefit mosquitoes by increasing the habitat availability for larvae [52]. Given concerted attempts to increase irrigation (e.g. NEPAD's Irrigation Programme, http://www.nepad-caadp.net) [53], there will be future trade-offs between increases in crop production, with the concomitant increases in food availability and income, and the negative effects of increased disease burden on humans, cattle and other livestock. Similar processes are likely underlying the positive relationship seen with higher RVF risk and cultivation, with the water needed even for non-irrigated lands providing good habitat for larval development. The increasing risk associated with greater numbers of people most likely reflects locations with more farming, more local irrigation and more local water resources for people and livestock to use as a daily water source. The surprising negative relationship between RVF risk and rainfall may be caused by the interaction of increased agricultural land-use and high human populations occurring in historically grassland areas with lower rainfall. Areas with higher rainfall, principally forested areas of Africa, have yet to be converted in earnest into agriculture.

The overall higher risk seen in strong ENSO event years (ONI > 1 or ONI < −1) is worrying, given a predicted doubling in the rate of ENSO events in the near future [54]. Understanding, for instance, why there is increased risk in South Africa in both El Niño and La Niña event years will be key, with the clear differences in areas that are at risk given opposing ONI scores. In strong La Niña event years, with commonly cooler and wetter climates, outbreaks appear to occur in the Karoo biomes, whereas in the dryer and warmer El Niño years, larger outbreaks occur in the central grassland biomes of South Africa. If increasing events do occur in the future, further research into these subtleties will be key in taking sound preventive measures.

Indeed, future models could explicitly incorporate expected global changes. Previous attempts with simple disease transmission models have approximated how host species are likely to change their distribution with upcoming climate and land-use change [3]. The ENSO modelling here suggests that both ‘warmer and drier’ or ‘cooler and wetter’ options have the potential to increase RVF cases, providing testable hypotheses about how predicted climate might impact future cases. Our results show that the risk of RVF varies as an interaction between space and time. There appears a limited need to undertake surveillance in, for instance, South Africa other than between December and March. It would appear prudent to prioritize monitoring in western Africa in non-categorized event (non-ENSO) years, South Africa, Kenya, Tanzania and Uganda in strong El Niño event years, and South Africa and Madagascar in strong La Niña event years. We highlight three areas that are potentially at high RVF risk but have historically very low reporting rates, namely southwestern Niger, western Chad and in the southeast of Sudan, especially in non-ENSO event and La Niña years. These countries have a history of political instability and low governance scores [55], and there may have been RVF cases present but limited infrastructure to diagnose and report them. These areas correspond to predicted areas of higher than expected sero-prevalence [56] and should be explored as possible risk locations for future outbreaks.

By understanding more about the structure of the data underlying RVF and accounting for spatial–temporal dynamics we have demonstrated an interpretable approach that could be applied to other diseases [12]. This might be especially useful to those at early stages of research such as the many neglected tropical diseases in Africa, southeast Asia and South America where detailed survey data are not available. As implemented in R-INLA package, INLA-based Bayesian models are fast, flexible and more interpretable for non-experts to implement [48]. More detailed risk models, such as the one we present here for RVF, can aid health planning to offset the impacts of debilitating diseases that disproportionately affect some of the poorest communities in the world.

## Supplementary Material

Supplementary tables and figures

## Data accessibility

The datasets supporting this article have been uploaded as part of the electronic supplementary material.
