# Supplementary material for: Spatial, seasonal and climatic predictive models of Rift Valley fever disease across Africa
Source: Philos Trans R Soc Lond B Biol Sci. 2017 Jun 5;372(1725):20160165. doi: 10.1098/rstb.2016.0165 (PMC5468690; doi:10.1098/rstb.2016.0165)
Supplement: Supplementary tables and figures [file rstb20160165supp1.docx]

ESM - Spatial, seasonal and climatic predictive models of Rift Valley Fever disease across Africa

David W. Redding^1*^, Sonia Tiedt^2^, Gianni Lo Iacono^3^, Bernard Bett^4^, and Kate E. Jones^5*^

^1^David W. Redding, Centre for Biodiversity and Environment Research, Department of Genetics, Evolution and Environment, University College London, Gower Street, London, WC1E 6BT, United Kingdom.

^2^Sonia Tiedt, Centre for Biodiversity and Environment Research, Department of Genetics, Evolution and Environment, University College London, Gower Street, London, WC1E 6BT, United Kingdom.

^3^Gianni Lo Iacono, Department of Veterinary Medicine, Disease Dynamics Unit, University of Cambridge, Madingley Road, Cambridge, CB3 0ES, United Kingdom, and Environmental Change, Public Health England, Didcot, Oxfordshire, OX11 0RQ, United Kingdom.

^4^Bernard Bett, International Livestock Research Institute, P. O. Box 30709-00100, Nairobi, Kenya.

^5^Kate E. Jones, Centre for Biodiversity and Environment Research, Department of Genetics, Evolution and Environment, University College London, Gower Street, London, WC1E 6BT, United Kingdom, and Institute of Zoology, Zoological Society of London, Regent’s Park, London, NW1 4RY, United Kingdom. 0000-0001-5231-3293.

* Corresponding authors: [dwredding@gmail.com](mailto:dwredding@gmail.com) and [kate.e.jones@ucl.ac.uk](mailto:kate.e.jones@ucl.ac.uk) (Tel: +44 (0)20 31084230)

**Table S1.** Details of confirmed RVF outbreaks from EMPRES-i database from 2004-2016 (n = 976). Co-ordinates are in decimal degrees. Each line represents a single case, multiple cases, or no cases found for livestock at that location. Sum at risk is an estimate of the animals at the location that could have contracted the disease and Q represents seasonal periods where Quarter 1 represents the period January to March; Quarter 2 April to June; Quarter 3 July to September; and Quarter 4 (October-December).

| Locations | Latitude | Longitude | Date | Q | Sum At Risk |
| --- | --- | --- | --- | --- | --- |
| Senegal, Saint Louis, Ross-Bathio | 16.3064 | -16.1256 | 01/01/2004 | 1 | NA |
| Mali, Sikasso, Yanfolila | 11.17 | -8.159 | 01/07/2004 | 3 | NA |
| Mali, Sikasso, Selingue | 11.619 | -8.241 | 01/07/2004 | 3 | NA |
| Mali, Mopti, Bofara | 14.181 | -4.184 | 01/07/2004 | 3 | NA |
| Senegal, Saint Louis, Mpal | 15.9571 | -16.2526 | 01/07/2004 | 3 | NA |
| Senegal, Saint Louis, Galoya | 16.05456 | -13.8568 | 01/07/2004 | 3 | NA |
| Senegal, Matam, Thilogne | 16.063 | -13.4371 | 01/07/2004 | 3 | NA |
| Mauritania, Assaba, Tijikja | 15.936 | -11.564 | 01/08/2004 | 3 | NA |
| Mauritania, Assaba, Kiffa | 16.634 | -11.341 | 01/08/2004 | 3 | NA |
| Mauritania, Tagant, Kankossa | 17.844 | -11.638 | 01/08/2004 | 3 | NA |
| Kenya, Rift Valley, Samburu District Rift Valley Kenya | 1.25 | 37 | 01/09/2004 | 3 | NA |
| Kenya, Western, Busia District Western Kenya | 0.416666 | 34.25 | 01/09/2004 | 3 | NA |
| Kenya, Coast, Malindi District Coast Province Kenya | -3.23333 | 40.1 | 01/09/2004 | 3 | NA |
| Mauritania, Assaba, Tijikja | 15.936 | -11.564 | 01/10/2004 | 4 | NA |
| Senegal, Louga, Kangaledji Pond Ferlo Region Senegal | 15.27361 | -14.8444 | 01/11/2004 | 4 | 59 |
| Senegal, Louga, Barkedji Pond Ferlo Region Senegal | 15.28333 | -14.8667 | 01/11/2004 | 4 | 40 |
| United Republic Of Tanzania, Tanga, Tanga United Republic Of Tanzania | -5.06667 | 39.1 | 01/11/2004 | 4 | NA |
| Senegal, Louga, Ngao Pond Ferlo Region Senegal | 15.24167 | -14.8514 | 01/11/2004 | 4 | 51 |
| Senegal, Louga, Furdu Pond Ferlo Region Senegal | 15.25583 | -14.8606 | 01/11/2004 | 4 | 30 |
| Senegal, Louga, Niaka Pond Ferlo Region Senegal | 15.29611 | -14.8989 | 01/11/2004 | 4 | 40 |
| Senegal, Saint Louis, Dagana | 16.24497 | -15.8867 | 01/11/2004 | 4 | NA |
| Senegal, Saint Louis, Keur Mbaye | 16.51 | -15.5 | 01/01/2005 | 1 | NA |
| Senegal, Tambacounda, Kidira | 14.46 | -12.21 | 01/08/2005 | 3 | NA |
| Mauritania, Guidimakha, Selibabi | 15.219 | -12.197 | 01/08/2005 | 3 | NA |
| Mauritania, Hodh Ech Chargi, Diguenni | 15.724 | -8.716 | 01/08/2005 | 3 | NA |
| Mauritania, Assaba, Tijikja | 15.936 | -11.564 | 01/08/2005 | 3 | NA |
| Senegal, Saint Louis, Mpal | 15.9571 | -16.2526 | 01/08/2005 | 3 | NA |
| Senegal, Saint Louis, Ross-Bathio | 16.3064 | -16.1256 | 01/08/2005 | 3 | NA |
| Mauritania, Hodh El Gharbi, Kobenni | 16.38 | -10.241 | 01/08/2005 | 3 | NA |
| Senegal, Saint Louis, Thilla Baoubacar | 16.5604 | -14.6861 | 01/08/2005 | 3 | NA |
| Mauritania, Hodh Ech Chargi, Nama | 16.624 | -7.362 | 01/08/2005 | 3 | NA |
| Mauritania, Assaba, Kiffa | 16.634 | -11.341 | 01/08/2005 | 3 | NA |
| Mauritania, Brakna, Boghe | 16.675 | -14.328 | 01/08/2005 | 3 | NA |
| Mauritania, Tagant, Kankossa | 17.844 | -11.638 | 01/08/2005 | 3 | NA |
| Senegal, Tambacounda, Kidira | 14.46 | -12.21 | 01/09/2005 | 3 | NA |
| Senegal, Saint Louis, Mpal | 15.9571 | -16.2526 | 01/09/2005 | 3 | NA |
| Senegal, Saint Louis, Ross-Bathio | 16.3064 | -16.1256 | 01/09/2005 | 3 | NA |
| Senegal, Saint Louis, Thilla Baoubacar | 16.5604 | -14.6861 | 01/09/2005 | 3 | NA |
| Mauritania, Hodh El Gharbi, Kobenni | 16.38 | -10.241 | 01/10/2005 | 4 | NA |
| Mauritania, Hodh Ech Chargi, Nama | 16.624 | -7.362 | 01/10/2005 | 4 | NA |
| Kenya, North Eastern, Adadi Jole | 3.33 | 39.46 | 01/12/2006 | 4 | NA |
| Kenya, Coast, Diani Konamusa | -4.43 | 39.454 | 04/12/2006 | 4 | NA |
| Kenya, Eastern, Karurumo | -0.476 | 37.667 | 04/12/2006 | 4 | NA |
| Kenya, Eastern, Tara | -1.238 | 37.317 | 04/12/2006 | 4 | NA |
| Kenya, Rift Valley, Giathugu | -1.591 | 36.935 | 04/12/2006 | 4 | NA |
| Kenya, Coast, Lamu | -2.185 | 40.383 | 04/12/2006 | 4 | NA |
| Kenya, Rift Valley, Marula | -0.879 | 36.196 | 04/12/2006 | 4 | NA |
| Kenya, North Eastern, Hola | 1.234 | 39.941 | 04/12/2006 | 4 | NA |
| Kenya, North Eastern, Masalani | -1.634 | 40.566 | 04/12/2006 | 4 | NA |
| Kenya, Eastern, Ngare Ndare | 0.187 | 37.427 | 04/12/2006 | 4 | NA |
| Kenya, Nyanza, Karia | -0.204 | 34.343 | 04/12/2006 | 4 | NA |
| Kenya, Nairobi, Kasarani | -1.22 | 36.9 | 04/12/2006 | 4 | NA |
| Kenya, Eastern, Baibariu | 0.293 | 38.084 | 04/12/2006 | 4 | NA |
| Kenya, Central, Ruiru | -1.131 | 36.915 | 04/12/2006 | 4 | NA |
| Kenya, Central, Njoguini Rurii | -0.67 | 37.179 | 04/12/2006 | 4 | NA |
| Kenya, North Eastern, Dadaab Galmagara Bura Shantabak Mbalambala Danyiri | 0.044 | 40.28 | 04/12/2006 | 4 | NA |
| Kenya, Eastern, Evurore Karaba Wachoro Mutito | -0.621 | 37.88 | 04/12/2006 | 4 | NA |
| Kenya, Coast, Mwatate Sisal Lualenyi Ranch Taveta | -3.389 | 37.672 | 04/12/2006 | 4 | NA |
| Kenya, North Eastern, Adadi Jole | 3.33 | 39.46 | 04/12/2006 | 4 | NA |
| Kenya, North Eastern, Dantu Kutulo Gari Didkuro | 3.43 | 40.968 | 04/12/2006 | 4 | NA |
| Kenya, Coast, Kilifi | -3.349 | 39.908 | 04/12/2006 | 4 | NA |
| Kenya, Coast, Kinango Musiu Lunga Lunga Kidzumbani | -4.546 | 39.121 | 04/12/2006 | 4 | NA |
| Kenya, North Eastern, Galmagara | -1.08 | 40.7 | 04/12/2006 | 4 | 27000 |
| Kenya, North Eastern, Liboi | 0.35278 | 40.86944 | 21/12/2006 | 4 | NA |
| Kenya, North Eastern, Baraki | 0.4925 | 39.51167 | 21/12/2006 | 4 | NA |
| Kenya, North Eastern, Shimbirey | 0.4925 | 39.83417 | 21/12/2006 | 4 | NA |
| Kenya, North Eastern, Jara Jila | 0.49583 | 40.83639 | 21/12/2006 | 4 | NA |
| Kenya, North Eastern, Shanta Abak | 0.5 | 39.5 | 21/12/2006 | 4 | NA |
| Kenya, North Eastern, Korakora | -0.5 | 39.83333 | 21/12/2006 | 4 | NA |
| Kenya, North Eastern, Fafi | 0.58444 | 40.34056 | 21/12/2006 | 4 | NA |
| Kenya, North Eastern, Garissa (Shimbirey) | -0.25094 | 40.25357 | 25/12/2006 | 4 | NA |
| Somalia, Juba Hoose, Bandar Jadiid | 0.01138 | 42.7033 | 01/01/2007 | 1 | NA |
| Kenya, Coast, Mombasa | -4.008 | 39.669 | 04/01/2007 | 1 | NA |
| Kenya, Coast, Lango Baya Viriku | -3.119 | 39.786 | 04/01/2007 | 1 | NA |
| Kenya, Eastern, Tharaka District Eastern Province Kenya | -0.3 | 38.01667 | 15/01/2007 | 1 | 81 |
| Kenya, Coast, Kwale District Coast Province Kenya | -4.16667 | 39.16667 | 15/01/2007 | 1 | 51 |
| Kenya, Eastern, Machakos District Eastern Province Kenya | -2 | 37.66667 | 15/01/2007 | 1 | 125 |
| Uganda, Mubende, Mbeere District Eastern Province Kenya | 0.566667 | 31.61667 | 15/01/2007 | 1 | 113 |
| Kenya, Eastern, Isiolo District Eastern Province Kenya | 1 | 38.75 | 15/01/2007 | 1 | 42 |
| Kenya, Eastern, Embu District Eastern Province Kenya | -0.58333 | 37.66667 | 15/01/2007 | 1 | 95 |
| Kenya, Coast, Taita District Coast Province Kenya | -3.5 | 38.5 | 15/01/2007 | 1 | 360 |
| Kenya, Eastern, Meru Central (District) Eatsern Province Kenya | 0.047035 | 37.6498 | 15/01/2007 | 1 | 42 |
| Kenya, North Eastern, Mandera District Northeastern Province Kenya | 3.5 | 40.75 | 15/01/2007 | 1 | 68 |
| Kenya, Rift Valley, Kajiado District Rift Valley Province Kenya | -2 | 36.75 | 15/01/2007 | 1 | 125 |
| Kenya, Rift Valley, Nakuru District Rift Valley Province Kenya | -0.33333 | 36 | 15/01/2007 | 1 | 176 |
| Kenya, Central, Thika District Central Province Kenya | -1.08333 | 37.08333 | 15/01/2007 | 1 | 52 |
| Kenya, Central, Kenol | -0.845 | 37.052 | 22/01/2007 | 1 | NA |
| Kenya, Eastern, Kavuti Ikime Malawa | -0.655 | 38.453 | 22/01/2007 | 1 | NA |
| Kenya, Eastern, Kauvi Kathyaka Ntahnge | -2.225 | 37.738 | 23/01/2007 | 1 | NA |
| Kenya, Eastern, Ikanga Ndetani Mailku Kyoani Ngungi Kituti | -1.69 | 38.108 | 23/01/2007 | 1 | NA |
| Kenya, Rift Valley, Nanyuki | 0.017 | 37.06 | 25/01/2007 | 1 | NA |
| Kenya, Coast, Gadoma | -3.372 | 39.304 | 29/01/2007 | 1 | NA |
| Kenya, Eastern, Town Ship | -0.315 | 37.62 | 31/01/2007 | 1 | NA |
| Kenya, Central, Gatundu Gikambura | -1.271 | 36.643 | 02/02/2007 | 1 | NA |
| Kenya, Central, Tebere | -0.666 | 37.36 | 02/02/2007 | 1 | NA |
| Kenya, Rift Valley, Longewan Sindani Kiserian Maji Ndege | 0.483 | 36.057 | 02/02/2007 | 1 | NA |
| Kenya, Rift Valley, Olot | 1.175 | 35.667 | 05/02/2007 | 1 | NA |
| Kenya, Rift Valley, Nodnyo Laresoro Lerata Sereolipi | 1.13 | 37.609 | 05/02/2007 | 1 | NA |
| United Republic Of Tanzania, Arusha, Makiba | -3.51897 | 36.96912 | 07/02/2007 | 1 | 32972 |
| United Republic Of Tanzania, Manyara, Galapo | -4.26667 | 35.85 | 07/02/2007 | 1 | NA |
| United Republic Of Tanzania, Tabora, Ifakara | -5.65 | 32.75 | 07/02/2007 | 1 | NA |
| United Republic Of Tanzania, Pwani, Saleni | -6.4064 | 38.38307 | 07/02/2007 | 1 | 32972 |
| United Republic Of Tanzania, Pwani, Makombe | -6.41083 | 38.29242 | 07/02/2007 | 1 | 32972 |
| United Republic Of Tanzania, Pwani, Mindutulieni | -6.45935 | 38.29723 | 07/02/2007 | 1 | 32972 |
| United Republic Of Tanzania, Pwani, Kalolenilulenge | -6.57155 | 38.12757 | 07/02/2007 | 1 | 32972 |
| United Republic Of Tanzania, Pwani, Buyuni | -6.60373 | 38.6416 | 07/02/2007 | 1 | 32972 |
| United Republic Of Tanzania, Pwani, Visakazi Juu | -6.62307 | 38.0664 | 07/02/2007 | 1 | 32972 |
| United Republic Of Tanzania, Pwani, Ruvu | -6.6818 | 38.64497 | 07/02/2007 | 1 | 32972 |
| United Republic Of Tanzania, Pwani, Matuli Mapalam | -6.68822 | 38.22757 | 07/02/2007 | 1 | 32972 |
| United Republic Of Tanzania, Pwani, Chamakweza | -6.69217 | 38.6177 | 07/02/2007 | 1 | 32972 |
| United Republic Of Tanzania, Pwani, Vigwaza | -6.69217 | 38.61922 | 07/02/2007 | 1 | 32972 |
| United Republic Of Tanzania, Morogoro, Kiberege | -7.97508 | 36.88097 | 07/02/2007 | 1 | 32972 |
| United Republic Of Tanzania, Morogoro, Samaganga | -8.07545 | 36.79525 | 07/02/2007 | 1 | 32972 |
| United Republic Of Tanzania, Morogoro, Lugongole | -8.09345 | 36.77773 | 07/02/2007 | 1 | 32972 |
| United Republic Of Tanzania, Pwani, Muhoro | -8.09995 | 38.20353 | 07/02/2007 | 1 | 32972 |
| United Republic Of Tanzania, Arusha, Makiba | -3.51897 | 36.96912 | 07/02/2007 | 1 | NA |
| Kenya, Rift Valley, Baringo District Rift Valley Province Kenya | 0.633333 | 36 | 01/03/2007 | 1 | NA |
| Kenya, Coast, Kilifi District Coast Province Kenya | -2.71667 | 40.2 | 01/03/2007 | 1 | NA |
| United Republic Of Tanzania, Arusha, Arusha | -2.91919 | 36.09845 | 23/03/2007 | 1 | NA |
| United Republic Of Tanzania, Manyara, Manyara | -4.68713 | 37.14545 | 23/03/2007 | 1 | NA |
| United Republic Of Tanzania, Tanga, Tanga | -5.06627 | 38.29217 | 23/03/2007 | 1 | NA |
| United Republic Of Tanzania, Dodoma, Dodoma | -5.84704 | 35.95974 | 23/03/2007 | 1 | NA |
| United Republic Of Tanzania, Morogoro, Morogoro | -7.86302 | 37.31286 | 23/03/2007 | 1 | NA |
| United Republic Of Tanzania, Mwanza, Mwanza Region Tanzania | -2.75 | 32.75 | 12/04/2007 | 2 | NA |
| United Republic Of Tanzania, Lindi, Lindi Region Tanzania | -9.5 | 38.5 | 12/04/2007 | 2 | NA |
| United Republic Of Tanzania, Mtwara, Mtwara Region Tanzania | -10.6667 | 39 | 18/04/2007 | 2 | NA |
| United Republic Of Tanzania, Mbeya, Mbeya Region Tanzania | -8.5 | 33 | 18/04/2007 | 2 | NA |
| United Republic Of Tanzania, Tanga, Tanga Region Tanzania | -5 | 38.25 | 09/05/2007 | 2 | NA |
| United Republic Of Tanzania, Iringa, Iringa District Iringa Rural Iringa Tanzania | -7.58333 | 35.5 | 15/06/2007 | 2 | NA |
| Sudan, White Nile, Zilait | 13.127 | 32.735 | 08/10/2007 | 4 | 510 |
| Sudan, White Nile, Zilait | 13.2 | 32.62 | 08/10/2007 | 4 | 510 |
| Sudan, Sennar, Sennar | 12.91812 | 34.13519 | 05/11/2007 | 4 | NA |
| Madagascar, Sava, Antsiranana | -13.4423 | 49.71916 | 01/01/2008 | 1 | NA |
| South Africa, Mpumalanga, Ngwenya | -25.395 | 31.84583 | 14/01/2008 | 1 | 371 |
| South Africa, Mpumalanga, Ten Bosch | -25.4936 | 31.80472 | 15/01/2008 | 1 | NA |
| Madagascar, Ihorombe, Fianarantsoa | -22.3795 | 45.80403 | 01/02/2008 | 1 | NA |
| South Africa, Mpumalanga, Vyeboom | -25.4342 | 31.8275 | 01/02/2008 | 1 | NA |
| Madagascar, Analamanga, Avaradrano | -18.91 | 47.51 | 04/02/2008 | 1 | 9 |
| South Africa, Mpumalanga, Martolhi | -25.4269 | 31.77139 | 07/02/2008 | 1 | 147 |
| South Africa, Mpumalanga, Grootboom | -25.427 | 31.7714 | 07/02/2008 | 1 | 316 |
| South Africa, Mpumalanga, Richtersnek | -25.6253 | 31.64944 | 12/02/2008 | 1 | NA |
| South Africa, Mpumalanga, One Tree Hill | -25.5427 | 31.6491 | 27/02/2008 | 1 | 462 |
| Madagascar, Atsinanana, Toamasina | -18.1239 | 49.344 | 01/03/2008 | 1 | NA |
| South Africa, Gauteng, Pretoria Metropolitan Council | -25.852 | 28.206 | 01/03/2008 | 1 | NA |
| South Africa, Limpopo, Langkuil | -25.0139 | 28.24306 | 01/03/2008 | 1 | NA |
| South Africa, Limpopo, Buiskop | -24.8606 | 28.3372 | 01/03/2008 | 1 | 300 |
| South Africa, Limpopo, Paul | -24.0351 | 31.0653 | 07/03/2008 | 1 | 60 |
| South Africa, Limpopo, Grietjie | -24.0969 | 31.0132 | 07/03/2008 | 1 | 80 |
| South Africa, Mpumalanga, Leeuwkraal | -25.1456 | 31.77139 | 07/03/2008 | 1 | 140 |
| Madagascar, Melaky, Mahajanga | -17.7417 | 44.39656 | 01/04/2008 | 2 | NA |
| Western Sahara, Saguia El Hamra, Meharrize | 26.14722 | -11.0692 | 01/04/2008 | 2 | 84 |
| Western Sahara, Saguia El Hamra, Bir Lehlu Saguia El Hamra Western Sahara | 26.30028 | -9.61056 | 01/04/2008 | 2 | 90 |
| South Africa, Limpopo, Leewdoorns | -24.8508 | 28.5075 | 01/04/2008 | 2 | NA |
| Western Sahara, Saguia El Hamra, Tifariti | 26.0928 | -10.6089 | 01/04/2008 | 2 | 89 |
| Algeria, Tindouf, Tindouf Province Algeria | 27.68333 | -8.13333 | 01/04/2008 | 2 | 459 |
| South Africa, Limpopo, Krokodildrift | -24.6667 | 27.66666 | 01/04/2008 | 2 | NA |
| South Africa, Mpumalanga, Doomkloof | -25.427 | 31.7714 | 07/04/2008 | 2 | 430 |
| Madagascar, Alaotra Mangoro, Alaotra Mangoro | -17.9242 | 48.32175 | 18/04/2008 | 2 | NA |
| Madagascar, Analamanga, Analamanga | -18.4051 | 47.4117 | 18/04/2008 | 2 | NA |
| Madagascar, Itasy, Itasy | -18.9277 | 46.81157 | 18/04/2008 | 2 | NA |
| Madagascar, Vakinankaratra, Vakinankaratra | -19.8921 | 46.68989 | 18/04/2008 | 2 | NA |
| Madagascar, Anosy, Anosy | -24.51 | 46.40802 | 18/04/2008 | 2 | NA |
| South Africa, Gauteng, Witpoort | -25.9722 | 28.55417 | 22/04/2008 | 2 | 150 |
| South Africa, North West, Mamagalieskraal | -25.5333 | 27.78333 | 23/04/2008 | 2 | 50 |
| South Africa, North West, Krokodildrift | -25.6667 | 27.76667 | 23/04/2008 | 2 | 73 |
| South Africa, North West, Leeuwkraal | -25.1667 | 28.05 | 23/04/2008 | 2 | 321 |
| Madagascar, Sofia, Mahajanga | -16.047 | 48.72116 | 01/05/2008 | 2 | NA |
| South Africa, Mpumalanga, Curlews Settlement | -25.3444 | 31.05556 | 01/05/2008 | 2 | NA |
| South Africa, Mpumalanga, Hooggelegen | -25.3594 | 31.02444 | 01/05/2008 | 2 | NA |
| South Africa, Gauteng, Witpoort | -25.9672 | 28.55417 | 01/05/2008 | 2 | NA |
| South Africa, Mpumalanga, Vaalbank | -25.1197 | 28.8375 | 01/05/2008 | 2 | NA |
| South Africa, Mpumalanga, Blinkwater | -25.3467 | 31.07639 | 01/05/2008 | 2 | NA |
| South Africa, Mpumalanga, Doornkloof | -25.426 | 31.77 | 04/05/2008 | 2 | NA |
| Madagascar, Menabe, Toliary | -20.5503 | 44.33844 | 01/06/2008 | 2 | NA |
| United Republic Of Tanzania, Mbeya, Mbeya Range United Republic Of Tanzania | -8.83333 | 33.33333 | 01/06/2008 | 2 | NA |
| Swaziland, Manzini, Serec | -26.4998 | 31.20274 | 04/06/2008 | 2 | 170 |
| Mali, Sikasso, Selingue | 11.619 | -8.241 | 01/07/2008 | 3 | NA |
| Madagascar, Atsimo Andrefana, Toliary | -22.8953 | 44.45234 | 01/07/2008 | 3 | NA |
| Mayotte, Administrative Unit Not Available, Combani | -12.784 | 45.141 | 11/07/2008 | 3 | 30 |
| Mauritania, Guidimakha, Selibabi | 15.219 | -12.197 | 01/08/2008 | 3 | NA |
| Mauritania, Assaba, Tijikja | 15.936 | -11.564 | 01/08/2008 | 3 | NA |
| Mauritania, Gorgol, Mbout | 16.012 | -12.522 | 01/08/2008 | 3 | NA |
| Mauritania, Hodh El Gharbi, Kobenni | 16.38 | -10.241 | 01/08/2008 | 3 | NA |
| Mauritania, Assaba, Kiffa | 16.634 | -11.341 | 01/08/2008 | 3 | NA |
| Mauritania, Brakna, Boghe | 16.675 | -14.328 | 01/08/2008 | 3 | NA |
| Madagascar, Boeny, Mahajanga | -16.6992 | 46.53728 | 01/08/2008 | 3 | NA |
| Mauritania, Trarza, R'kiz | 16.836 | -15.341 | 01/08/2008 | 3 | NA |
| Mauritania, Tagant, Kankossa | 17.844 | -11.638 | 01/08/2008 | 3 | NA |
| Madagascar, Atsimo Andrefana, Toliary | -23.2942 | 43.70534 | 01/08/2008 | 3 | NA |
| Madagascar, Atsinanana, Toraka Malagasy Madagascar | -18.3506 | 49.32312 | 01/08/2008 | 3 | 4426 |
| Swaziland, Manzini, Mccreedy | -26.486 | 31.192 | 12/08/2008 | 3 | 69 |
| Madagascar, Alaotra Mangoro, Toamasina | -17.8648 | 48.37958 | 01/09/2008 | 3 | NA |
| Madagascar, Atsimo Andrefana, Toliary | -23.0678 | 43.80467 | 01/09/2008 | 3 | NA |
| Mauritania, Hodh El Gharbi, Kobenni | 16.38 | -10.241 | 01/10/2008 | 4 | NA |
| Mauritania, Assaba, Kiffa | 16.634 | -11.341 | 01/10/2008 | 4 | NA |
| Mauritania, Brakna, Boghe | 16.675 | -14.328 | 01/10/2008 | 4 | NA |
| Mauritania, Tagant, Kankossa | 17.844 | -11.638 | 01/10/2008 | 4 | NA |
| Madagascar, Atsimo Andrefana, Toliary | -24.6123 | 44.41089 | 01/10/2008 | 4 | NA |
| Madagascar, Diana, Antsiranana | -12.5417 | 49.16687 | 01/11/2008 | 4 | NA |
| Madagascar, Haute Matsiatra, Nasandratrony | -21.7167 | 46.9333 | 16/11/2008 | 4 | NA |
| Madagascar, Haute Matsiatra, Antady | -21.3833 | 46.1333 | 16/11/2008 | 4 | 9 |
| Madagascar, Haute Matsiatra, Ambanimaso | -21.3667 | 46.55 | 22/11/2008 | 4 | 9 |
| Madagascar, Haute Matsiatra, Imandry | -21.3778 | 46.66 | 24/11/2008 | 4 | 8 |
| Madagascar, Haute Matsiatra, Tsaramandroso | -21.3079 | 46.2313 | 29/11/2008 | 4 | 9 |
| Senegal, Matam, Ourosogui | 15.6396 | -13.3101 | 01/12/2008 | 4 | NA |
| Senegal, Saint Louis, Mpal | 15.9571 | -16.2526 | 01/12/2008 | 4 | NA |
| Senegal, Saint Louis, Keur Mbaye | 16.51 | -15.5 | 01/12/2008 | 4 | NA |
| Senegal, Saint Louis, Thilla Baoubacar | 16.5604 | -14.6861 | 01/12/2008 | 4 | NA |
| Mauritania, Brakna, Boghe | 16.675 | -14.328 | 01/12/2008 | 4 | NA |
| Madagascar, Menabe, Toliary | -19.6722 | 44.79772 | 01/12/2008 | 4 | NA |
| Madagascar, Haute Matsiatra, Antanifotsy | -21.37 | 46.14 | 10/12/2008 | 4 | 7 |
| Madagascar, Haute Matsiatra, Ampasina | -21.35 | 46.81 | 11/12/2008 | 4 | 3 |
| Madagascar, Haute Matsiatra, Ambonifehidrano | -21.39 | 46.25 | 13/12/2008 | 4 | 2 |
| Madagascar, Haute Matsiatra, Marodinta | -21.75 | 46.92 | 13/12/2008 | 4 | 1 |
| Uganda, Masaka, Ndyamuba Farm | -0.35665 | 31.7216 | 01/01/2009 | 1 | 45 |
| Uganda, Ssembabule, Nagadya Farm | -0.02319 | 31.33961 | 01/01/2009 | 1 | 40 |
| Uganda, Masaka, Kezimbira Farm | -0.35665 | 31.7216 | 01/01/2009 | 1 | 122 |
| Uganda, Masaka, Tugume Farm | -0.35665 | 31.7216 | 01/01/2009 | 1 | 85 |
| Uganda, Ssembabule, Nakabale Farm | -0.02319 | 31.33961 | 01/01/2009 | 1 | 40 |
| Uganda, Masaka, Mukuye Farm | -0.35665 | 31.7216 | 01/01/2009 | 1 | 70 |
| Uganda, Mubende, Peace Farm | 0.516436 | 31.56608 | 01/01/2009 | 1 | 30 |
| Uganda, Ssembabule, Ssembeguya Farm | -0.02319 | 31.33961 | 01/01/2009 | 1 | 120 |
| Uganda, Ssembabule, Katumba Farm Sembabule Uganda | -0.02319 | 31.33961 | 01/01/2009 | 1 | 30 |
| Uganda, Mubende, Brund Enterprise Farm Mubende Uganda | 0.516436 | 31.56608 | 01/01/2009 | 1 | 70 |
| Uganda, Gomba, Mayigaharriet Farm | 0.13935 | 31.93705 | 01/01/2009 | 1 | 23 |
| Uganda, Ssembabule, Lubega Farm | -0.02319 | 31.33961 | 01/01/2009 | 1 | 20 |
| Uganda, Mubende, Hb Ranch Farm | 0.516436 | 31.56608 | 01/01/2009 | 1 | 50 |
| Uganda, Masaka, Mugula Farm Masaka Uganda | -0.35665 | 31.7216 | 01/01/2009 | 1 | 103 |
| Uganda, Gomba, Lydiabigo Farm | 0.13935 | 31.93705 | 01/01/2009 | 1 | 55 |
| Uganda, Masaka, Sheikh Kasumba Farm | -0.35665 | 31.7216 | 01/01/2009 | 1 | 84 |
| Uganda, Mubende, Kisombwa Farm | 0.516436 | 31.56608 | 01/01/2009 | 1 | 210 |
| Uganda, Gomba, Mujabi Farm | 0.13935 | 31.93705 | 01/01/2009 | 1 | 50 |
| Uganda, Gomba, Maduwa Farm | 0.13935 | 31.93705 | 01/01/2009 | 1 | 80 |
| Uganda, Gomba, Katabarwa Farm | 0.13935 | 31.93705 | 01/01/2009 | 1 | 96 |
| Uganda, Gomba, Kadd Farm | 0.13935 | 31.93705 | 01/01/2009 | 1 | 104 |
| Uganda, Gomba, Mutagubya Farm | 0.13935 | 31.93705 | 01/01/2009 | 1 | 179 |
| Uganda, Gomba, Kisakye Farm | 0.13935 | 31.93705 | 01/01/2009 | 1 | 200 |
| Uganda, Gomba, Hamidu Mayanja Farm Mpigi Uganda | 0.13935 | 31.93705 | 01/01/2009 | 1 | 187 |
| South Africa, Kwazulu-Natal, Stockton | -29.947 | 29.986 | 18/02/2009 | 1 | 800 |
| South Africa, Kwazulu-Natal, Tyrone | -29.882 | 30.018 | 18/02/2009 | 1 | 1000 |
| South Africa, Kwazulu-Natal, Ingwe | -29.0306 | 29.88695 | 23/02/2009 | 1 | NA |
| South Africa, Kwazulu-Natal, Kwa Sam | -29.1636 | 29.85742 | 23/02/2009 | 1 | NA |
| South Africa, Kwazulu-Natal, Kwa Sam | -29.5448 | 29.80374 | 23/02/2009 | 1 | NA |
| South Africa, Kwazulu-Natal, Ingwe | -29.8239 | 30.02383 | 23/02/2009 | 1 | NA |
| South Africa, Kwazulu-Natal, Ingwe | -29.9527 | 29.99699 | 23/02/2009 | 1 | NA |
| South Africa, Kwazulu-Natal, Ingwe | -29.9715 | 29.97283 | 23/02/2009 | 1 | NA |
| South Africa, Kwazulu-Natal, Ingwe | -29.9984 | 29.95136 | 23/02/2009 | 1 | NA |
| South Africa, Kwazulu-Natal, Allandale | -29.955 | 29.969 | 25/02/2009 | 1 | 100 |
| South Africa, Kwazulu-Natal, Conference | -30.021 | 29.886 | 25/02/2009 | 1 | 500 |
| South Africa, Kwazulu-Natal, Glen Gowrey | -29.85 | 29.229 | 27/02/2009 | 1 | NA |
| South Africa, Kwazulu-Natal, Eastwold | -29.994 | 29.947 | 27/02/2009 | 1 | 800 |
| South Africa, Kwazulu-Natal, Ringstead | -29.796 | 29.535 | 03/03/2009 | 1 | 1000 |
| South Africa, Kwazulu-Natal, Mount Hermon | -30.1667 | 29.93333 | 09/03/2009 | 1 | 600 |
| South Africa, Kwazulu-Natal, Burnview | -30.0167 | 29.83333 | 23/03/2009 | 1 | 800 |
| South Africa, Mpumalanga, Mbombela Rural | -25.3077 | 31.11048 | 01/05/2009 | 2 | NA |
| Madagascar, Analamanga, Anjozorobe Analamanga Antananarivo Madagascar | -18.4 | 47.86667 | 01/05/2009 | 2 | 894 |
| South Africa, Kwazulu-Natal, Lions River | -29.4636 | 30.16469 | 04/06/2009 | 2 | NA |
| South Africa, Northern Cape, Zwaardraai | -28.6696 | 20.47217 | 14/10/2009 | 4 | 256 |
| South Africa, Northern Cape, Rooipad G | -28.6347 | 20.30083 | 20/10/2009 | 4 | 15 |
| South Africa, Northern Cape, Soetap | -28.7839 | 20.68417 | 20/10/2009 | 4 | 118 |
| South Africa, Northern Cape, Augrabies Klint | -28.6011 | 20 | 20/10/2009 | 4 | 1734 |
| South Africa, Northern Cape, Daliah | -28.7011 | 20.33361 | 20/10/2009 | 4 | 19 |
| South Africa, Northern Cape, Johnsies | -28.5398 | 20.53058 | 20/10/2009 | 4 | 107 |
| South Africa, Northern Cape, Witkopeiland A | -28.7333 | 20.51694 | 20/10/2009 | 4 | 115 |
| South Africa, Northern Cape, Tweestrome | -28.7003 | 20.61806 | 20/10/2009 | 4 | 188 |
| South Africa, Northern Cape, Witkopeiland Vw | -28.7169 | 20.51722 | 20/10/2009 | 4 | 16 |
| South Africa, Northern Cape, Kobbe Kobbe | -28.7342 | 20.51694 | 20/10/2009 | 4 | 8 |
| South Africa, Northern Cape, Neusberg | -28.7667 | 20.71667 | 20/10/2009 | 4 | 250 |
| South Africa, Northern Cape, Shallom | -28.8006 | 20.55 | 20/10/2009 | 4 | 24 |
| South Africa, Northern Cape, Becker Farm | -28.6686 | 20.43111 | 20/10/2009 | 4 | 5 |
| South Africa, Northern Cape, Keimoes | -28.6344 | 21.05111 | 20/10/2009 | 4 | 12 |
| South Africa, Northern Cape, Vaas Kraal | -28.4675 | 20.20028 | 20/10/2009 | 4 | 6500 |
| South Africa, Northern Cape, Rooipad | -28.4833 | 20.33361 | 20/10/2009 | 4 | 57 |
| South Africa, Northern Cape, Augrabies | -28.6333 | 20.3675 | 20/10/2009 | 4 | 160 |
| South Africa, Northern Cape, Bakenrand | -28.6333 | 20.43417 | 20/10/2009 | 4 | 150 |
| South Africa, Northern Cape, Kannoneiland | -28.6591 | 21.1 | 02/12/2009 | 4 | 20 |
| Sudan, Al Jazeera, El Gezira State Al Jazeera Sudan | 14.61044 | 33.3522 | 01/01/2010 | 1 | NA |
| South Africa, Free State, Gegund | -28.219 | 26.28878 | 19/01/2010 | 1 | 350 |
| South Africa, Free State, Meyerspruit | -29.4741 | 26.3022 | 01/02/2010 | 1 | 1008 |
| South Africa, Free State, Rotterdam | -27.0966 | 27.6588 | 04/02/2010 | 1 | 40 |
| South Africa, Free State, Eldorado | -28.1791 | 26.4797 | 07/02/2010 | 1 | 200 |
| South Africa, Free State, Willowmore | -28.1811 | 26.30502 | 11/02/2010 | 1 | 110 |
| South Africa, Free State, Helderfontein | -28.4653 | 26.385 | 12/02/2010 | 1 | 750 |
| South Africa, Free State, Patryskloof | -28.5633 | 26.38055 | 12/02/2010 | 1 | 797 |
| South Africa, Free State, Vaalbank | -27.8372 | 26.58777 | 14/02/2010 | 1 | 145 |
| South Africa, Free State, Rietvlei | -27.6875 | 25.77361 | 14/02/2010 | 1 | 297 |
| South Africa, Eastern Cape, Vetfontein | -31.2697 | 25.20056 | 15/02/2010 | 1 | 21 |
| South Africa, Free State, Boskoppie | -27.7431 | 27.2275 | 15/02/2010 | 1 | NA |
| South Africa, Free State, Kleinbegin | -28.3736 | 26.4708 | 15/02/2010 | 1 | 52 |
| South Africa, Free State, Tonning | -29.2165 | 24.88883 | 15/02/2010 | 1 | 200 |
| South Africa, Free State, Geskenk | -27.6831 | 26.18194 | 15/02/2010 | 1 | 200 |
| South Africa, Free State, Vlaklaagte | -27.7711 | 26.63306 | 15/02/2010 | 1 | 55 |
| South Africa, Free State, Nelspan | -28.3258 | 26.64306 | 15/02/2010 | 1 | 96 |
| South Africa, Free State, Maatskappy | -27.9359 | 26.92233 | 15/02/2010 | 1 | 230 |
| South Africa, Free State, Doringdeel | -28.3196 | 26.875 | 15/02/2010 | 1 | 65 |
| South Africa, Free State, Klipheuwel | -27.8347 | 26.4169 | 15/02/2010 | 1 | 180 |
| South Africa, Free State, Deelpan | -27.8786 | 26.36056 | 15/02/2010 | 1 | 203 |
| South Africa, Free State, Smaldraai | -28.2182 | 26.44195 | 15/02/2010 | 1 | 120 |
| South Africa, Free State, Salmienas Rust | -28.6136 | 26.28361 | 15/02/2010 | 1 | 530 |
| South Africa, Free State, Vaalkoppies | -28.1419 | 26.43306 | 15/02/2010 | 1 | 194 |
| South Africa, Free State, Paradys | -28.5128 | 26.27389 | 15/02/2010 | 1 | 1727 |
| South Africa, Free State, Hagenstad | -28.7136 | 26.08694 | 16/02/2010 | 1 | 300 |
| South Africa, Free State, Erfdeel | -28.0235 | 25.88958 | 16/02/2010 | 1 | 500 |
| South Africa, Free State, Talla | -28.4722 | 26.425 | 16/02/2010 | 1 | 680 |
| South Africa, Free State, Klipkuil | -28.2408 | 26.15417 | 17/02/2010 | 1 | 400 |
| South Africa, Free State, Schoonspruit | -27.6319 | 26.59278 | 17/02/2010 | 1 | 340 |
| South Africa, Free State, Mooipan | -28.2801 | 26.63438 | 17/02/2010 | 1 | 350 |
| South Africa, Free State, Doring Draai | -28.7961 | 26.33694 | 18/02/2010 | 1 | 509 |
| South Africa, Free State, Kalkdam | -28.3613 | 26.11494 | 18/02/2010 | 1 | 566 |
| South Africa, Free State, Jupiter | -27.8817 | 26.40025 | 18/02/2010 | 1 | 220 |
| South Africa, Free State, Normandi | -28.45 | 26.41667 | 18/02/2010 | 1 | 36 |
| South Africa, Free State, Fairview | -27.8083 | 26.5666 | 18/02/2010 | 1 | 167 |
| South Africa, Free State, Waterput | -29.6635 | 24.71259 | 18/02/2010 | 1 | 5200 |
| South Africa, Free State, Coetzeevlei | -28.1952 | 26.44144 | 18/02/2010 | 1 | 40 |
| South Africa, Free State, Blydschap | -27.8769 | 28.28389 | 19/02/2010 | 1 | 170 |
| South Africa, Free State, Goedemoed | -28.0812 | 26.25561 | 19/02/2010 | 1 | 30 |
| South Africa, Northern Cape, Taaiboschfontein | -30.7186 | 24.71614 | 19/02/2010 | 1 | 4504 |
| South Africa, Free State, Magdalena | -27.8544 | 26.35467 | 19/02/2010 | 1 | 420 |
| South Africa, Free State, Electi | -28.1509 | 26.26183 | 19/02/2010 | 1 | 370 |
| South Africa, Free State, Vreugde | -29.0542 | 27.13 | 19/02/2010 | 1 | 200 |
| South Africa, Free State, Kortlaagte | -27.9395 | 26.53311 | 19/02/2010 | 1 | 297 |
| South Africa, Free State, Stillerust | -28.5853 | 26.1625 | 19/02/2010 | 1 | 2200 |
| South Africa, Free State, Roodevallei | -28.429 | 26.22233 | 19/02/2010 | 1 | 450 |
| South Africa, Free State, Rondavel | -28.4281 | 26.41167 | 19/02/2010 | 1 | 1758 |
| South Africa, Free State, Weltevrede | -28.4072 | 26.25889 | 19/02/2010 | 1 | 418 |
| South Africa, Free State, Adriana | -28.2923 | 26.12219 | 20/02/2010 | 1 | 143 |
| South Africa, Free State, Kalkfontein | -27.6291 | 27.0425 | 20/02/2010 | 1 | 670 |
| South Africa, Free State, Vrede | -27.0902 | 27.095 | 20/02/2010 | 1 | 1900 |
| South Africa, Free State, Dawie | -28.0511 | 26.2186 | 20/02/2010 | 1 | 380 |
| South Africa, Free State, Thelma | -27.8714 | 26.72222 | 20/02/2010 | 1 | 403 |
| South Africa, Free State, Deelpan - Geluk | -28.627 | 26.298 | 20/02/2010 | 1 | 700 |
| South Africa, Free State, Annasdal | -28.625 | 25.5916 | 20/02/2010 | 1 | 225 |
| South Africa, Free State, Ratau | -29.2353 | 26.08278 | 22/02/2010 | 1 | 12 |
| South Africa, Free State, Kruidfontein | -29.5964 | 25.7333 | 22/02/2010 | 1 | 684 |
| South Africa, Free State, Modderfontein | -27.7737 | 26.53567 | 22/02/2010 | 1 | 1110 |
| South Africa, Free State, Onssin | -28.1639 | 26.41694 | 22/02/2010 | 1 | 2000 |
| South Africa, Free State, Goliatskraal | -27.8277 | 27.06969 | 23/02/2010 | 1 | 16 |
| South Africa, Free State, Eikelaan Bainsvlei | -29.0575 | 26.10768 | 23/02/2010 | 1 | 31 |
| South Africa, Free State, London | -27.204 | 28.74353 | 23/02/2010 | 1 | 417 |
| South Africa, Free State, Geluk | -28.4892 | 25.66611 | 23/02/2010 | 1 | 470 |
| South Africa, Free State, Groenvlei | -29.0613 | 26.1675 | 25/02/2010 | 1 | 98 |
| South Africa, Free State, Holfontein | -28.7169 | 25.94611 | 25/02/2010 | 1 | 58 |
| South Africa, Free State, Ventersvlei | -29.4694 | 25.94833 | 25/02/2010 | 1 | 188 |
| South Africa, Free State, Kromdraai | -29.6655 | 25.96111 | 25/02/2010 | 1 | 2040 |
| South Africa, Free State, Geduld | -28.3625 | 26.1161 | 25/02/2010 | 1 | 800 |
| South Africa, Free State, Mahemsfontein | -28.4236 | 25.43861 | 25/02/2010 | 1 | 1400 |
| South Africa, Free State, Doornlaagte | -28.8694 | 25.41306 | 25/02/2010 | 1 | 704 |
| South Africa, Free State, Aurora | -28.0125 | 28.76611 | 26/02/2010 | 1 | 330 |
| South Africa, Free State, Kleinplaats | -29.7422 | 26.1983 | 26/02/2010 | 1 | 850 |
| South Africa, Free State, Delportsrus | -29.9431 | 26.61333 | 26/02/2010 | 1 | 775 |
| South Africa, Free State, Hartbeespan | -29.1763 | 25.1348 | 27/02/2010 | 1 | 600 |
| South Africa, Free State, Hillarydale | -27.6292 | 27.0425 | 28/02/2010 | 1 | 420 |
| South Africa, Free State, Albertina | -27.7533 | 26.85472 | 28/02/2010 | 1 | 162 |
| South Africa, Free State, Klipkuil | -28.3861 | 26.10556 | 28/02/2010 | 1 | 42 |
| South Africa, Free State, Zaaiplaats | -27.2058 | 27.43194 | 28/02/2010 | 1 | 1000 |
| Madagascar, Analamanga, Anjozorobe -Angavo (Last Outbreak: 2008-2009) | -18.3116 | 48.016 | 01/03/2010 | 1 | 1610 |
| South Africa, Eastern Cape, Romansfontein | -31.5167 | 26.23333 | 01/03/2010 | 1 | 2300 |
| South Africa, Western Cape, Breede River District Council (Adm. Lev. 2 Name) | -33.744 | 19.535 | 01/03/2010 | 1 | NA |
| South Africa, Free State, Mooidam | -26.9533 | 27.773 | 01/03/2010 | 1 | 67 |
| South Africa, Free State, Vlaklaagte Suid | -27.253 | 28.0769 | 01/03/2010 | 1 | 235 |
| South Africa, Free State, Verdien | -27.919 | 27.296 | 01/03/2010 | 1 | 1500 |
| South Africa, Free State, Ouwerf | -28.0133 | 26.23944 | 01/03/2010 | 1 | 600 |
| South Africa, Free State, Hope Valley | -29.202 | 26.1679 | 01/03/2010 | 1 | 73 |
| South Africa, Free State, Vadersgift | -28.2108 | 26.52361 | 01/03/2010 | 1 | 180 |
| South Africa, Gauteng, Ystervarkfontein | -26.042 | 28.54778 | 01/03/2010 | 1 | 229 |
| South Africa, Free State, Brakpan | -28.0354 | 26.50139 | 01/03/2010 | 1 | 45 |
| South Africa, Free State, Glen Ross | -28.1537 | 26.73308 | 01/03/2010 | 1 | 167 |
| South Africa, Free State, Geluksdeel | -29.2567 | 25.87833 | 01/03/2010 | 1 | 365 |
| South Africa, Northern Cape, Springbokpoortjie | -30.1246 | 22.32919 | 01/03/2010 | 1 | 1400 |
| South Africa, Free State, Helka | -28.0497 | 25.9733 | 01/03/2010 | 1 | 605 |
| South Africa, Free State, Grasslands | -28.9216 | 26.1661 | 01/03/2010 | 1 | 800 |
| South Africa, Eastern Cape, Groot Dam Farm 1 | -31.7833 | 25.7 | 01/03/2010 | 1 | 4580 |
| South Africa, Free State, Wattle Grove | -28.1739 | 28.22761 | 02/03/2010 | 1 | 30 |
| South Africa, Northern Cape, Smart Sindikaat | -30.5571 | 23.53583 | 02/03/2010 | 1 | 1373 |
| South Africa, Free State, Danielskuil | -27.5764 | 26.36639 | 02/03/2010 | 1 | 519 |
| South Africa, Northern Cape, Tzamenkomst | -30.775 | 24.83056 | 03/03/2010 | 1 | 700 |
| South Africa, Free State, Lesparance | -27.8264 | 27.08777 | 03/03/2010 | 1 | 110 |
| South Africa, Free State, Schoongesicht | -29.5831 | 25.81333 | 03/03/2010 | 1 | 2000 |
| South Africa, Free State, Free State | -28.6814 | 26.62748 | 04/03/2010 | 1 | NA |
| South Africa, Northern Cape, Northern Cape | -28.8484 | 22.02168 | 04/03/2010 | 1 | NA |
| South Africa, Northern Cape, Wimbledon | -29.1086 | 23.90889 | 04/03/2010 | 1 | 606 |
| South Africa, Free State, Preithos | -29.1161 | 25.38639 | 04/03/2010 | 1 | 140 |
| South Africa, Eastern Cape, Beaconsfield | -31.5792 | 25.53139 | 04/03/2010 | 1 | 1000 |
| South Africa, Free State, Brakpoort | -27.9219 | 26.4475 | 04/03/2010 | 1 | 100 |
| South Africa, Eastern Cape, Hillston | -31.3976 | 25.49567 | 05/03/2010 | 1 | 2000 |
| South Africa, Free State, Malloch | -29.4797 | 26.0022 | 05/03/2010 | 1 | 292 |
| South Africa, Northern Cape, Plum | -29.2221 | 23.57011 | 05/03/2010 | 1 | 600 |
| South Africa, Free State, Mooivlakte | -29.0255 | 26.1191 | 05/03/2010 | 1 | 156 |
| South Africa, Free State, Bosdam | -29.3327 | 25.9252 | 05/03/2010 | 1 | 300 |
| South Africa, Free State, Palmietfontein | -29.7581 | 25.87194 | 05/03/2010 | 1 | 1520 |
| South Africa, Northern Cape, Remhoogte | -29.5278 | 23.00917 | 05/03/2010 | 1 | 790 |
| South Africa, Free State, Doornbult | -27.7488 | 25.8648 | 06/03/2010 | 1 | 264 |
| South Africa, Free State, Welgelegen | -29.3731 | 24.58012 | 06/03/2010 | 1 | 800 |
| South Africa, Free State, Vermont | -29.9641 | 26.594 | 07/03/2010 | 1 | 2100 |
| South Africa, Free State, Edaneg | -29.1839 | 24.79834 | 07/03/2010 | 1 | 78 |
| South Africa, Free State, Ragnarok | -28.3228 | 26.07361 | 07/03/2010 | 1 | 93 |
| South Africa, Gauteng, Bossemanskraal | -25.8883 | 28.85444 | 08/03/2010 | 1 | 270 |
| South Africa, Northern Cape, Kleinfontein | -31.1167 | 25.2 | 08/03/2010 | 1 | 3383 |
| South Africa, Free State, Bankfontein | -27.4388 | 28.1163 | 08/03/2010 | 1 | 300 |
| South Africa, Northern Cape, Roodepan | -28.64 | 24.72865 | 08/03/2010 | 1 | 49 |
| South Africa, Mpumalanga, Silverbank | -26.9 | 28.78333 | 08/03/2010 | 1 | 150 |
| South Africa, Eastern Cape, Paardekraal | -31.3972 | 26.37556 | 08/03/2010 | 1 | 64 |
| South Africa, Mpumalanga, Bosjespruit | -26.9667 | 28.81667 | 08/03/2010 | 1 | 660 |
| South Africa, Northern Cape, De Jagersvlei | -29.7827 | 22.907 | 08/03/2010 | 1 | 1002 |
| South Africa, Free State, Majaki Trust | -27.5219 | 27.39008 | 09/03/2010 | 1 | 50 |
| South Africa, North West, Potchefstroom Experimental Farm | -26.7686 | 26.99344 | 09/03/2010 | 1 | 1097 |
| South Africa, Free State, Vrede | -27.8882 | 27.08903 | 09/03/2010 | 1 | 1900 |
| South Africa, Northern Cape, Kenhardt Meent | -29.3092 | 21.1473 | 09/03/2010 | 1 | 2500 |
| South Africa, Eastern Cape, Wolwekop | -31.3567 | 25.01744 | 09/03/2010 | 1 | 1500 |
| South Africa, Eastern Cape, Helpmekaar | -31.53 | 25.55908 | 09/03/2010 | 1 | 5000 |
| South Africa, Free State, Toch Gekry | -28.9313 | 25.6805 | 10/03/2010 | 1 | NA |
| South Africa, Free State, Daskop | -27.2273 | 27.50905 | 10/03/2010 | 1 | NA |
| South Africa, Free State, Cecilia | -29.215 | 26.1336 | 10/03/2010 | 1 | 99 |
| South Africa, Free State, The Chase | -29.0633 | 25.72306 | 10/03/2010 | 1 | 136 |
| South Africa, Free State, Goedehoop | -29.0506 | 25.53083 | 10/03/2010 | 1 | 375 |
| South Africa, Free State, Geluk 2484 | -27.7369 | 27.0133 | 10/03/2010 | 1 | 1200 |
| South Africa, Free State, Lovedale | -28.9314 | 25.68056 | 11/03/2010 | 1 | 300 |
| South Africa, North West, Schoonheid | -27.8434 | 25.09168 | 11/03/2010 | 1 | 100 |
| South Africa, Mpumalanga, Witpoort | -26.7167 | 28.8 | 11/03/2010 | 1 | 250 |
| South Africa, Free State, Uithoek | -28.9538 | 26.1019 | 11/03/2010 | 1 | 450 |
| South Africa, Free State, Anex Twyfelpoort | -29.3867 | 25.1286 | 11/03/2010 | 1 | 146 |
| South Africa, Free State, Homerule | -28.9306 | 25.52 | 11/03/2010 | 1 | 280 |
| South Africa, Free State, Platkop | -28.92 | 25.3 | 12/03/2010 | 1 | 316 |
| South Africa, Northern Cape, Abrahamsvlei | -30.6294 | 20.88865 | 12/03/2010 | 1 | 500 |
| South Africa, Eastern Cape, Blaauwskop | -31.5886 | 25.56025 | 12/03/2010 | 1 | 150 |
| South Africa, Northern Cape, Wildebeestkuil | -28.7161 | 24.69806 | 12/03/2010 | 1 | 150 |
| South Africa, Free State, Roodewal | -29.195 | 26.6097 | 12/03/2010 | 1 | 40 |
| South Africa, Free State, Klippan | -27.0716 | 27.3216 | 12/03/2010 | 1 | 228 |
| South Africa, Free State, Laugh Dearg | -28.9163 | 25.8691 | 12/03/2010 | 1 | 92 |
| South Africa, Free State, Raadzel | -29.0828 | 25.22972 | 12/03/2010 | 1 | 550 |
| South Africa, Northern Cape, Kameelkolk | -30.8625 | 20.74722 | 12/03/2010 | 1 | 320 |
| South Africa, Free State, Legkraal | -29.3658 | 25.48944 | 12/03/2010 | 1 | 2000 |
| South Africa, Free State, Damfontein | -28.9858 | 25.26917 | 12/03/2010 | 1 | 750 |
| South Africa, Free State, Donkerhoek | -28.8756 | 26.13222 | 13/03/2010 | 1 | 1148 |
| South Africa, Free State, Glen Lyon | -28.9783 | 26.2997 | 13/03/2010 | 1 | 1397 |
| South Africa, Northern Cape, Slingerfontein | -30.8167 | 25.25 | 13/03/2010 | 1 | 2800 |
| South Africa, Eastern Cape, Leeuwfontein | -31.2342 | 25.99139 | 14/03/2010 | 1 | 1350 |
| South Africa, Northern Cape, Jan Se Boom | -30.8093 | 21.5204 | 14/03/2010 | 1 | 1125 |
| South Africa, Free State, Duraville | -28.831 | 24.83101 | 14/03/2010 | 1 | 300 |
| South Africa, Free State, De Rust | -27.7597 | 26.8719 | 15/03/2010 | 1 | 195 |
| South Africa, Free State, Teboho Trust | -27.8633 | 27.65889 | 15/03/2010 | 1 | 100 |
| South Africa, Free State, Bainsvlei | -29.0813 | 26.1269 | 15/03/2010 | 1 | 45 |
| South Africa, Free State, Alandale | -27.6241 | 29.22854 | 15/03/2010 | 1 | 1500 |
| South Africa, Free State, Roodeheuwel | -28.8394 | 26.4444 | 15/03/2010 | 1 | 177 |
| South Africa, Free State, Klein Morgenson | -29.288 | 25.9188 | 15/03/2010 | 1 | 90 |
| South Africa, Free State, Weltevrede | -29.503 | 24.9104 | 15/03/2010 | 1 | NA |
| South Africa, Free State, Emmerentia | -27.921 | 28.557 | 15/03/2010 | 1 | 650 |
| South Africa, Free State, Roodepoort | -29.7302 | 25.9911 | 15/03/2010 | 1 | 1500 |
| South Africa, Northern Cape, Kleinplaas | -29.9744 | 24.62247 | 15/03/2010 | 1 | 301 |
| South Africa, Northern Cape, Platfontein | -28.6643 | 24.60981 | 15/03/2010 | 1 | 50 |
| South Africa, Northern Cape, Grasberg | -31.0695 | 23.1827 | 15/03/2010 | 1 | 249 |
| South Africa, Free State, Brabant | -29.0457 | 26.0508 | 15/03/2010 | 1 | 39 |
| South Africa, Free State, Mooigenoeg | -29.1231 | 25.21972 | 15/03/2010 | 1 | 320 |
| South Africa, Northern Cape, Kraankuil | -29.8813 | 24.17719 | 15/03/2010 | 1 | 40 |
| South Africa, Free State, Wildealskloof | -29.0188 | 26.2353 | 15/03/2010 | 1 | 1900 |
| South Africa, Free State, Hoekpan | -29.1464 | 25.38556 | 15/03/2010 | 1 | 129 |
| South Africa, Free State, Vaaldan | -29.4713 | 24.6218 | 15/03/2010 | 1 | 332 |
| South Africa, Northern Cape, Klerefontein | -30.971 | 21.9815 | 15/03/2010 | 1 | 1268 |
| South Africa, Northern Cape, Fairfield | -28.3616 | 24.329 | 15/03/2010 | 1 | 176 |
| South Africa, Free State, Biesieput | -29.1203 | 25.24 | 15/03/2010 | 1 | 203 |
| South Africa, Free State, Gruisdam | -29.3867 | 25.12862 | 15/03/2010 | 1 | 654 |
| South Africa, Free State, Koppieskraal | -29.2936 | 24.9669 | 15/03/2010 | 1 | 820 |
| South Africa, Free State, Caledonsdraai | -28.6149 | 28.084 | 15/03/2010 | 1 | 286 |
| South Africa, Free State, Gaanspan | -29.2472 | 24.8683 | 15/03/2010 | 1 | 200 |
| South Africa, Free State, Brakfontein | -29.8078 | 26.13056 | 15/03/2010 | 1 | 1500 |
| South Africa, Northern Cape, Glen Allen | -29.6512 | 22.61031 | 15/03/2010 | 1 | 1070 |
| South Africa, Free State, Equusbult | -29.2916 | 24.60894 | 15/03/2010 | 1 | 1996 |
| South Africa, Free State, Kroonvlei | -27.3525 | 27.2527 | 15/03/2010 | 1 | 550 |
| South Africa, Free State, Magpela | -30.2444 | 26.6973 | 15/03/2010 | 1 | 1000 |
| South Africa, Northern Cape, Luckhoff | -30.7374 | 22.10308 | 15/03/2010 | 1 | 550 |
| South Africa, North West, Rooihoogte | -27.4267 | 25.99028 | 16/03/2010 | 1 | 57 |
| South Africa, Free State, Hebron | -29.005 | 25.9658 | 16/03/2010 | 1 | 158 |
| South Africa, Free State, Middelpunt | -28.9861 | 25.38806 | 16/03/2010 | 1 | 56 |
| South Africa, Northern Cape, Biesiespan | -30.5627 | 24.57408 | 16/03/2010 | 1 | 1116 |
| South Africa, Eastern Cape, Friesfontein | -31.4589 | 25.51512 | 16/03/2010 | 1 | 1500 |
| South Africa, Free State, Bellona | -28.49 | 27.359 | 16/03/2010 | 1 | 450 |
| South Africa, Free State, Welverdiend | -29.4067 | 25.00453 | 16/03/2010 | 1 | 420 |
| South Africa, Free State, Wintershoek | -29.3292 | 24.9329 | 16/03/2010 | 1 | 600 |
| South Africa, Northern Cape, Kleinbooi | -30.4761 | 21.57669 | 16/03/2010 | 1 | NA |
| South Africa, Free State, Felicitas | -29.6017 | 26.14056 | 16/03/2010 | 1 | NA |
| South Africa, Northern Cape, Paauwpan | -30.0387 | 24.155 | 17/03/2010 | 1 | 3000 |
| South Africa, Northern Cape, Leeukolk | -30.4218 | 21.29858 | 17/03/2010 | 1 | 600 |
| South Africa, Northern Cape, Papparaas | -30.5372 | 21.65097 | 17/03/2010 | 1 | 15 |
| South Africa, Eastern Cape, Temple Farm | -31.568 | 25.47203 | 17/03/2010 | 1 | 1500 |
| South Africa, Free State, Helena | -28.9877 | 26.0283 | 17/03/2010 | 1 | 32 |
| South Africa, Northern Cape, Melkdam | -30.461 | 22.13939 | 17/03/2010 | 1 | 162 |
| South Africa, Free State, Xavier | -29.1086 | 26.28 | 17/03/2010 | 1 | 120 |
| South Africa, Free State, Braklaagte | -29.2022 | 25.4111 | 17/03/2010 | 1 | 65 |
| South Africa, Northern Cape, Mierdam | -30.0842 | 22.31097 | 17/03/2010 | 1 | 801 |
| South Africa, Free State, Weltevreden | -29.3458 | 25.3333 | 17/03/2010 | 1 | 1500 |
| South Africa, Free State, Grootkuil | -28.2322 | 26.50806 | 17/03/2010 | 1 | 772 |
| South Africa, Northern Cape, De Nauwte | -30.1412 | 21.70478 | 17/03/2010 | 1 | 1250 |
| South Africa, Free State, Doringfontein | -29.4213 | 24.757 | 17/03/2010 | 1 | 327 |
| South Africa, Northern Cape, Gansvlei | -31.4143 | 22.02328 | 17/03/2010 | 1 | 554 |
| South Africa, Free State, Middenwater | -27.1719 | 28.6 | 18/03/2010 | 1 | 680 |
| South Africa, Free State, Harmony | -29.348 | 26.0858 | 18/03/2010 | 1 | 145 |
| South Africa, North West, Catharina | -28.062 | 25.005 | 18/03/2010 | 1 | 860 |
| South Africa, Free State, Geluk | -27.31 | 26.9733 | 18/03/2010 | 1 | 658 |
| South Africa, North West, North-West | -26.3711 | 25.23723 | 19/03/2010 | 1 | NA |
| South Africa, Eastern Cape, Eastern Cape | -32.1086 | 26.5905 | 19/03/2010 | 1 | NA |
| South Africa, Western Cape, Western Cape | -32.6322 | 22.22634 | 19/03/2010 | 1 | NA |
| South Africa, Free State, Springboklaagte | -27.6963 | 27.0261 | 19/03/2010 | 1 | 502 |
| South Africa, Eastern Cape, Kruisrivier | -30.9053 | 26.0625 | 19/03/2010 | 1 | 672 |
| South Africa, Eastern Cape, Beestekuil | -31.3033 | 25.35972 | 19/03/2010 | 1 | 100 |
| South Africa, Eastern Cape, Manor Home | -31.4867 | 25.46694 | 19/03/2010 | 1 | 1000 |
| South Africa, Free State, Windpoort | -30.5763 | 26.7566 | 19/03/2010 | 1 | 628 |
| South Africa, Western Cape, Hillandale | -31.9363 | 22.74928 | 19/03/2010 | 1 | 14 |
| South Africa, Eastern Cape, Dwarsvlei | -31.6369 | 25.00611 | 19/03/2010 | 1 | 2000 |
| South Africa, Free State, Bougainvillea | -29.7877 | 26.218 | 19/03/2010 | 1 | NA |
| South Africa, Northern Cape, Basterkraal | -32.0958 | 20.66167 | 19/03/2010 | 1 | 50 |
| South Africa, Free State, Tweelingspan | -28.9597 | 25.4666 | 19/03/2010 | 1 | 200 |
| South Africa, Northern Cape, Droehout | -29.968 | 21.431 | 19/03/2010 | 1 | 1200 |
| South Africa, Free State, Lilysdale | -28.4642 | 26.22694 | 19/03/2010 | 1 | 155 |
| South Africa, Free State, Hamelfontein | -28.615 | 26.86763 | 19/03/2010 | 1 | 4600 |
| South Africa, North West, Graslaagte | -26.5102 | 26.91567 | 20/03/2010 | 1 | 8 |
| South Africa, Free State, Groot Kallesfontein | -30.0608 | 24.8819 | 20/03/2010 | 1 | 2050 |
| South Africa, Free State, Lettiesrust | -27.052 | 27.612 | 20/03/2010 | 1 | 230 |
| South Africa, Free State, Buitendags Hoop | -27.802 | 26.7145 | 20/03/2010 | 1 | 70 |
| South Africa, Free State, Fonteinspruit | -27.5767 | 27.49333 | 20/03/2010 | 1 | 250 |
| South Africa, Free State, Bloedfontein | -30.0234 | 24.94342 | 21/03/2010 | 1 | 2050 |
| South Africa, Free State, Bankfontein | -30.0608 | 24.88194 | 21/03/2010 | 1 | 2050 |
| South Africa, Northern Cape, Koppiesdam | -29.9979 | 23.30089 | 21/03/2010 | 1 | NA |
| South Africa, Free State, Hertzog | -28.6636 | 26.33917 | 21/03/2010 | 1 | 1504 |
| South Africa, Eastern Cape, Skoongesig | -31.3519 | 25.44417 | 22/03/2010 | 1 | 500 |
| South Africa, Free State, Fontein | -27.8711 | 27.2175 | 22/03/2010 | 1 | 445 |
| South Africa, Northern Cape, Blaauwsyfer | -31.1267 | 21.36406 | 22/03/2010 | 1 | 500 |
| South Africa, Northern Cape, Ramkraal | -30.0513 | 22.94589 | 22/03/2010 | 1 | NA |
| South Africa, Northern Cape, Fonteintjie | -31.075 | 25.32389 | 23/03/2010 | 1 | 1700 |
| South Africa, Mpumalanga, Wolwefontein | -26.9608 | 28.71222 | 23/03/2010 | 1 | 680 |
| South Africa, Free State, Tweeling | -27.5413 | 28.5258 | 23/03/2010 | 1 | 900 |
| South Africa, Free State, Philadelphia | -27.9063 | 26.4318 | 23/03/2010 | 1 | 165 |
| South Africa, Northern Cape, Kloofsig | -29.956 | 24.56272 | 23/03/2010 | 1 | 1082 |
| South Africa, Northern Cape, Groot Markt | -30.2312 | 22.32431 | 23/03/2010 | 1 | 2000 |
| South Africa, Free State, Mijnrus | -29.719 | 26.6145 | 23/03/2010 | 1 | 702 |
| South Africa, Free State, Asbosdam | -29.1721 | 24.8658 | 23/03/2010 | 1 | 448 |
| South Africa, Free State, Denmark | -30.5938 | 26.8127 | 24/03/2010 | 1 | 740 |
| South Africa, Northern Cape, Stofkraalspoort | -31.1649 | 21.93178 | 24/03/2010 | 1 | NA |
| South Africa, Northern Cape, Poortjie | -31.4998 | 22.37633 | 24/03/2010 | 1 | 520 |
| South Africa, Northern Cape, Poortjies | -29.9772 | 21.31594 | 24/03/2010 | 1 | 500 |
| South Africa, Eastern Cape, Liebendal | -31.7179 | 25.92528 | 24/03/2010 | 1 | 800 |
| South Africa, Western Cape, Bleakhouse | -32.287 | 22.82 | 24/03/2010 | 1 | 2000 |
| South Africa, Northern Cape, Klein Lemoenkop-Wes | -29.9 | 20.7333 | 24/03/2010 | 1 | 1200 |
| South Africa, Northern Cape, Herbou | -29.6747 | 23.29608 | 24/03/2010 | 1 | 1184 |
| South Africa, Free State, Danzig | -29.1714 | 25.0754 | 24/03/2010 | 1 | NA |
| South Africa, Free State, Thorn Grove | -28.8352 | 26.1936 | 24/03/2010 | 1 | 800 |
| South Africa, Free State, De Wetsdrift | -27.1277 | 26.9525 | 25/03/2010 | 1 | 170 |
| South Africa, North West, Misgun Kareeboomput | -27.7083 | 25.05417 | 25/03/2010 | 1 | 110 |
| South Africa, Free State, Pleasure | -27.3452 | 27.21972 | 25/03/2010 | 1 | 428 |
| South Africa, Northern Cape, Taaibos | -29.5946 | 23.10206 | 25/03/2010 | 1 | 729 |
| South Africa, Free State, Leeuwkop | -29.4369 | 25.24944 | 25/03/2010 | 1 | 996 |
| South Africa, Free State, Blydskap | -27.2658 | 28.8425 | 26/03/2010 | 1 | 1120 |
| South Africa, Northern Cape, Liefdedal | -28.813 | 24.52542 | 26/03/2010 | 1 | 26 |
| South Africa, Free State, Rondefontein | -30.2283 | 25.69833 | 26/03/2010 | 1 | 147 |
| South Africa, Western Cape, Klipkraal | -32.0409 | 23.00822 | 26/03/2010 | 1 | 1016 |
| South Africa, Free State, Skoon Uitzight | -30.205 | 25.70333 | 26/03/2010 | 1 | 1048 |
| South Africa, Free State, Schulpspruit | -26.9614 | 27.3966 | 26/03/2010 | 1 | 41 |
| South Africa, Eastern Cape, Henningsfontein | -31.2725 | 25.40889 | 26/03/2010 | 1 | 1175 |
| South Africa, Free State, Slangfontein | -30.1861 | 25.56944 | 26/03/2010 | 1 | 1500 |
| South Africa, Northern Cape, Poloko Farm | -28.5774 | 24.02775 | 27/03/2010 | 1 | NA |
| South Africa, Northern Cape, Fonteintjie | -28.95 | 23.86667 | 27/03/2010 | 1 | 360 |
| South Africa, Northern Cape, De Put | -30.596 | 22.52788 | 27/03/2010 | 1 | 1267 |
| South Africa, Free State, Balmont | -30.4719 | 26.6697 | 28/03/2010 | 1 | 406 |
| South Africa, Free State, Goeie Hoop | -28.2259 | 28.04131 | 28/03/2010 | 1 | 270 |
| South Africa, Free State, Rooirand | -29.4738 | 25.39 | 28/03/2010 | 1 | 103 |
| South Africa, Eastern Cape, Mount Melsetter | -31.4428 | 25.47 | 28/03/2010 | 1 | 940 |
| South Africa, Northern Cape, Rooipan Droogedam | -29.9705 | 23.5525 | 28/03/2010 | 1 | 230 |
| South Africa, Free State, Viljoenskroon Townlands | -27.2183 | 27.9513 | 29/03/2010 | 1 | 299 |
| South Africa, Northern Cape, Fergus West | -29.2654 | 24.47086 | 29/03/2010 | 1 | 289 |
| South Africa, Free State, Kedron | -27.3914 | 27.01556 | 29/03/2010 | 1 | 244 |
| South Africa, Free State, Kalbasdrif | -29.5117 | 25.2925 | 29/03/2010 | 1 | 81 |
| South Africa, Northern Cape, Drie Plotte | -29.05 | 24.59361 | 29/03/2010 | 1 | NA |
| South Africa, Free State, Bultfontein Reddersburg | -29.5858 | 26.29444 | 29/03/2010 | 1 | 4100 |
| South Africa, Northern Cape, Oyfenhoudtsdrift Perseel 235 | -28.48 | 21.2403 | 29/03/2010 | 1 | 500 |
| South Africa, Western Cape, Sekretariskraal | -31.9347 | 23.51967 | 29/03/2010 | 1 | 100 |
| South Africa, Northern Cape, Groener Vlei | -28.5167 | 24.51667 | 30/03/2010 | 1 | 600 |
| South Africa, Northern Cape, Graspan | -29.3442 | 24.40819 | 30/03/2010 | 1 | 800 |
| South Africa, North West, Langerust - Oxendale | -26.45 | 24.56667 | 30/03/2010 | 1 | 22 |
| South Africa, Eastern Cape, Prospect | -31.8667 | 25.41667 | 30/03/2010 | 1 | 1820 |
| South Africa, Northern Cape, Orania Plot 29 | -29.8354 | 24.38117 | 30/03/2010 | 1 | 5 |
| South Africa, North West, Taaibospan | -26.5 | 25.75 | 31/03/2010 | 1 | 1 |
| South Africa, Northern Cape, Onrusfontein | -30.0197 | 24.43981 | 31/03/2010 | 1 | NA |
| South Africa, Eastern Cape, Buffelskop | -31.4833 | 25.11667 | 31/03/2010 | 1 | 26 |
| South Africa, Eastern Cape, Bangor | -31.3922 | 25.04783 | 31/03/2010 | 1 | 1500 |
| South Africa, Free State, Golden Valley | -27.3383 | 27.223 | 31/03/2010 | 1 | 605 |
| South Africa, Free State, Blaauwbank | -29.4688 | 25.10682 | 31/03/2010 | 1 | 376 |
| South Africa, Eastern Cape, The Pines | -31.5521 | 26.00358 | 31/03/2010 | 1 | 2630 |
| South Africa, Northern Cape, Smithdale | -28.4833 | 24.7166 | 31/03/2010 | 1 | 652 |
| South Africa, Free State, Hartebeesdam | -29.3931 | 24.56151 | 31/03/2010 | 1 | 235 |
| South Africa, Free State, Biesiesfontein | -29.56 | 24.75392 | 31/03/2010 | 1 | 716 |
| South Africa, Free State, Stofdam | -29.401 | 24.66005 | 31/03/2010 | 1 | 166 |
| South Africa, Free State, Palmietfontein | -29.2333 | 25.72444 | 31/03/2010 | 1 | 278 |
| South Africa, Free State, Uitzight | -29.515 | 24.81704 | 31/03/2010 | 1 | 744 |
| South Africa, Mpumalanga, Elim | -26.3075 | 29.84222 | 01/04/2010 | 2 | 425 |
| South Africa, Free State, Kromdraai | -27.7711 | 26.63306 | 01/04/2010 | 2 | 260 |
| South Africa, Northern Cape, Rietrivier | -29.11 | 24.5975 | 01/04/2010 | 2 | 110 |
| South Africa, Northern Cape, Perseel 3 M15 | -27.6617 | 24.70083 | 01/04/2010 | 2 | 61 |
| South Africa, Northern Cape, Geduld Boerdery | -28.7452 | 20.6054 | 01/04/2010 | 2 | 230 |
| South Africa, Northern Cape, Saaidam | -29.8485 | 23.3449 | 01/04/2010 | 2 | 2228 |
| South Africa, Northern Cape, Swemkuil | -29.4167 | 23.4167 | 01/04/2010 | 2 | 915 |
| South Africa, Western Cape, Coleshill | -32.3421 | 22.6283 | 02/04/2010 | 2 | 10 |
| South Africa, Northern Cape, Skietkolk | -30.8062 | 21.7816 | 02/04/2010 | 2 | 1522 |
| South Africa, Eastern Cape, Glencoe | -31.7768 | 25.30722 | 02/04/2010 | 2 | 2650 |
| South Africa, Eastern Cape, Odendal | -30.6811 | 26.7569 | 03/04/2010 | 2 | 9 |
| South Africa, Northern Cape, Rietrivier | -29.0973 | 24.60622 | 03/04/2010 | 2 | NA |
| South Africa, Eastern Cape, Keerom | -30.8172 | 25.6213 | 04/04/2010 | 2 | 950 |
| South Africa, Northern Cape, Doornkloof Portion 2 | -28.2083 | 24.48333 | 05/04/2010 | 2 | 230 |
| South Africa, Northern Cape, Adamsfontein | -30.5893 | 25.2227 | 05/04/2010 | 2 | 130 |
| South Africa, Northern Cape, Rietfontein | -30.6166 | 25.3333 | 05/04/2010 | 2 | 150 |
| South Africa, Northern Cape, Preservation | -29.7545 | 22.6913 | 05/04/2010 | 2 | 500 |
| South Africa, Northern Cape, Hydepark | -29.6695 | 22.8111 | 05/04/2010 | 2 | 398 |
| South Africa, Northern Cape, Steenbokkamp | -29.1001 | 24.60817 | 06/04/2010 | 2 | 84 |
| South Africa, Northern Cape, Tasmania | -29.6601 | 22.79225 | 06/04/2010 | 2 | 5 |
| South Africa, Northern Cape, Uitkyk | -30.2387 | 23.61122 | 06/04/2010 | 2 | NA |
| South Africa, Western Cape, Bakenskraal | -33.6378 | 22.21472 | 06/04/2010 | 2 | 300 |
| South Africa, Free State, Viljoenskroon Townlands | -27.2683 | 26.95138 | 06/04/2010 | 2 | 7 |
| South Africa, Free State, Drummond | -27.463 | 27.286 | 06/04/2010 | 2 | 400 |
| South Africa, Eastern Cape, Deochandoris | -31.4 | 27.6 | 06/04/2010 | 2 | 330 |
| South Africa, Free State, Rooiplaat | -29.5343 | 25.0375 | 06/04/2010 | 2 | 120 |
| South Africa, Free State, Hekkamp | -30.4161 | 26.975 | 06/04/2010 | 2 | 140 |
| South Africa, Eastern Cape, Panorama | -31.9969 | 25.49708 | 06/04/2010 | 2 | NA |
| South Africa, Free State, Leegtepoort | -29.3618 | 25.1271 | 06/04/2010 | 2 | 1400 |
| South Africa, Northern Cape, Daggafontein | -30.9166 | 23.2666 | 06/04/2010 | 2 | 2199 |
| South Africa, Western Cape, Snyderskraal | -31.8469 | 23.7384 | 07/04/2010 | 2 | 800 |
| South Africa, Free State, Vaalbank | -27.0772 | 28.4061 | 07/04/2010 | 2 | 1757 |
| South Africa, Free State, Poortjie | -29.715 | 24.58192 | 07/04/2010 | 2 | 462 |
| South Africa, Free State, Eensgevonden | -29.5676 | 25.06 | 07/04/2010 | 2 | 352 |
| South Africa, Northern Cape, Soutputs | -29.4211 | 22.2535 | 07/04/2010 | 2 | 550 |
| South Africa, Free State, Nalisview | -29.2352 | 26.2238 | 07/04/2010 | 2 | 158 |
| South Africa, Free State, Loskop | -29.4764 | 24.69575 | 07/04/2010 | 2 | 241 |
| South Africa, Free State, Winkelhaak | -29.4303 | 25.2058 | 07/04/2010 | 2 | 300 |
| South Africa, Free State, Vrykamp | -29.1141 | 24.7801 | 07/04/2010 | 2 | 312 |
| South Africa, Northern Cape, Alexandria | -31.3381 | 23.60938 | 07/04/2010 | 2 | 1125 |
| South Africa, Free State, Inkomst | -29.583 | 25.05211 | 07/04/2010 | 2 | 949 |
| South Africa, Free State, Brakput | -29.6404 | 24.66508 | 07/04/2010 | 2 | 1250 |
| South Africa, Free State, Kanonkop | -29.0175 | 24.9462 | 07/04/2010 | 2 | 225 |
| South Africa, Free State, De Kiel Oos | -29.3382 | 24.8213 | 07/04/2010 | 2 | 700 |
| South Africa, Free State, De Klei Oos | -29.3382 | 24.82137 | 07/04/2010 | 2 | 700 |
| South Africa, Northern Cape, Sanddrift | -31.1869 | 24.9519 | 08/04/2010 | 2 | 160 |
| South Africa, North West, Marotse Village | -26.75 | 25.5 | 08/04/2010 | 2 | 100 |
| South Africa, Northern Cape, Oudam | -29.4667 | 24.43314 | 08/04/2010 | 2 | 417 |
| South Africa, Free State, Wesselsdal | -30.0068 | 26.8873 | 08/04/2010 | 2 | 2653 |
| South Africa, Northern Cape, Oranjerivier Voere | -29.89 | 22.9795 | 08/04/2010 | 2 | 3412 |
| South Africa, Northern Cape, Ventersvallei | -30.3393 | 24.7661 | 09/04/2010 | 2 | 2033 |
| South Africa, Free State, Samekomst | -28.8861 | 25.4488 | 09/04/2010 | 2 | NA |
| South Africa, Northern Cape, Nuwekraal | -30.0765 | 23.6706 | 09/04/2010 | 2 | NA |
| South Africa, Free State, Holpan | -29.1609 | 24.666 | 09/04/2010 | 2 | 95 |
| South Africa, Free State, Wolwekop | -29.5483 | 24.8381 | 09/04/2010 | 2 | NA |
| South Africa, Free State, Rustplaas | -27.4289 | 27.2058 | 09/04/2010 | 2 | 363 |
| South Africa, Free State, Deellaagte | -29.4925 | 24.53306 | 09/04/2010 | 2 | 1000 |
| South Africa, Free State, Bosjespan | -29.6136 | 24.6227 | 09/04/2010 | 2 | NA |
| South Africa, Free State, Ferreira | -29.2022 | 26.1747 | 10/04/2010 | 2 | 16 |
| South Africa, Eastern Cape, Tweefontein | -31.5763 | 24.921 | 10/04/2010 | 2 | 1500 |
| South Africa, Eastern Cape, Landsdown | -31.6138 | 25.32972 | 10/04/2010 | 2 | 2000 |
| South Africa, Free State, Randfontein | -27.3769 | 26.575 | 10/04/2010 | 2 | 102 |
| South Africa, Free State, Tweefontein Fs | -29.5997 | 26.1027 | 10/04/2010 | 2 | 1000 |
| South Africa, Western Cape, Sonop | -33.8391 | 22.03386 | 11/04/2010 | 2 | 478 |
| South Africa, Northern Cape, Donnybrook | -29.5667 | 24.31667 | 11/04/2010 | 2 | 1520 |
| South Africa, Eastern Cape, Kalkoenkrantz | -31.55 | 26.3873 | 11/04/2010 | 2 | 250 |
| South Africa, Northern Cape, Modderfontein | -28.8484 | 22.02168 | 11/04/2010 | 2 | 1500 |
| South Africa, Eastern Cape, Terminus | -31.9046 | 25.4732 | 12/04/2010 | 2 | 1500 |
| South Africa, Free State, Bains Game Lodge | -29.0768 | 26.15137 | 12/04/2010 | 2 | NA |
| South Africa, Western Cape, Karee Bosch | -32.0155 | 23.38275 | 12/04/2010 | 2 | 300 |
| South Africa, Northern Cape, Perseel 2 Jx 4 | -27.7092 | 24.79378 | 12/04/2010 | 2 | 77 |
| South Africa, Northern Cape, Heuningneskloof | -29.1833 | 24.5 | 13/04/2010 | 2 | 3504 |
| South Africa, Northern Cape, Rhenosterfontein | -30.329 | 23.8961 | 13/04/2010 | 2 | NA |
| South Africa, Northern Cape, Kolkenburg | -30.6811 | 23.51628 | 13/04/2010 | 2 | 62 |
| South Africa, Northern Cape, Holpan | -30.428 | 23.19478 | 13/04/2010 | 2 | NA |
| South Africa, Free State, Pepershoek | -28.6146 | 26.86762 | 13/04/2010 | 2 | 825 |
| South Africa, Free State, Katfontein | -30.2688 | 26.0444 | 13/04/2010 | 2 | NA |
| South Africa, Eastern Cape, Experimental Farm Cradock | -32.22 | 25.6866 | 13/04/2010 | 2 | 3000 |
| South Africa, Eastern Cape, Strydomskraal | -31.9453 | 25.177 | 13/04/2010 | 2 | 1500 |
| South Africa, Northern Cape, Bakenkop | -28.9382 | 22.04696 | 13/04/2010 | 2 | 400 |
| South Africa, Northern Cape, Monterey | -29.5513 | 23.1296 | 13/04/2010 | 2 | 3525 |
| South Africa, Northern Cape, Grasbult | -28.125 | 24.8625 | 14/04/2010 | 2 | 1150 |
| South Africa, Northern Cape, Dombietersfontein | -31.0905 | 23.39108 | 14/04/2010 | 2 | NA |
| South Africa, Northern Cape, Ben Ora | -28.5087 | 21.1785 | 14/04/2010 | 2 | 250 |
| South Africa, Northern Cape, Christiaanskuil | -30.1412 | 23.9615 | 14/04/2010 | 2 | 265 |
| South Africa, Northern Cape, Klipdrift | -29.0794 | 24.59664 | 14/04/2010 | 2 | 312 |
| South Africa, Free State, Vredebloem | -27.9739 | 26.5681 | 14/04/2010 | 2 | 151 |
| South Africa, Free State, Blouboshoek | -30.0125 | 24.9275 | 14/04/2010 | 2 | NA |
| South Africa, Northern Cape, Tromps Graf | -31.263 | 22.91408 | 14/04/2010 | 2 | NA |
| South Africa, Northern Cape, Middelplaas | -28.1189 | 24.82483 | 15/04/2010 | 2 | 300 |
| South Africa, Northern Cape, Lieberwalt | -30.0026 | 24.6804 | 15/04/2010 | 2 | 294 |
| South Africa, Northern Cape, Komnader | -28.9596 | 23.9266 | 15/04/2010 | 2 | 131 |
| South Africa, Free State, Rosendal | -29.6869 | 26.2966 | 15/04/2010 | 2 | 720 |
| South Africa, Free State, Buonapartes Fontein | -30.5183 | 25.6427 | 15/04/2010 | 2 | 3500 |
| South Africa, Northern Cape, Standen | -28.8484 | 22.02168 | 15/04/2010 | 2 | 2000 |
| South Africa, Northern Cape, Magersfontein | -28.95 | 24.73333 | 16/04/2010 | 2 | 3810 |
| South Africa, Northern Cape, Buchlands | -29.059 | 23.6919 | 16/04/2010 | 2 | 307 |
| South Africa, Northern Cape, Witplaas | -30.6735 | 23.9823 | 16/04/2010 | 2 | 158 |
| South Africa, Western Cape, Courlands Kloof | -32.0833 | 22.9482 | 16/04/2010 | 2 | 2134 |
| South Africa, Free State, Kraaipoort | -29.4534 | 25.2495 | 16/04/2010 | 2 | 338 |
| South Africa, Northern Cape, Delportshoop Plot | -28.4167 | 24.28333 | 17/04/2010 | 2 | 62 |
| South Africa, Northern Cape, Bletterman | -30.7077 | 24.077 | 17/04/2010 | 2 | 83 |
| South Africa, Northern Cape, De Hoop | -28.8 | 24.5 | 17/04/2010 | 2 | 102 |
| South Africa, Eastern Cape, Katkop | -31.8874 | 25.4882 | 17/04/2010 | 2 | 1500 |
| South Africa, Free State, Memo | -29.416 | 24.51656 | 17/04/2010 | 2 | 772 |
| South Africa, Western Cape, Landskroon | -33.7592 | 18.9143 | 18/04/2010 | 2 | 301 |
| South Africa, Free State, Johannasrus | -27.0836 | 27.9833 | 19/04/2010 | 2 | 262 |
| South Africa, North West, Mietjiesdoorns | -27.2375 | 25.7558 | 19/04/2010 | 2 | NA |
| South Africa, Eastern Cape, Aarbossiesplaat | -31.1713 | 26.2088 | 19/04/2010 | 2 | 2100 |
| South Africa, Western Cape, Montainview | -34.0092 | 22.2725 | 20/04/2010 | 2 | 55 |
| South Africa, Western Cape, Toverwater | -32.0714 | 23.86333 | 20/04/2010 | 2 | 177 |
| South Africa, Northern Cape, Dysonsklip | -28.6167 | 21.1 | 20/04/2010 | 2 | 50 |
| South Africa, Northern Cape, Seekoeigat | -30.3451 | 23.7059 | 20/04/2010 | 2 | 800 |
| South Africa, North West, Goedgedacht | -26.5333 | 26.333 | 20/04/2010 | 2 | 550 |
| South Africa, Free State, Rooifontein | -28.8093 | 24.8624 | 22/04/2010 | 2 | NA |
| South Africa, Eastern Cape, Vrolikskraal | -31.7175 | 25.7494 | 22/04/2010 | 2 | 2000 |
| South Africa, Free State, West Wind Estate | -30.1822 | 25.7372 | 22/04/2010 | 2 | 4 |
| South Africa, Western Cape, Harmonie | -32.2351 | 23.64227 | 22/04/2010 | 2 | 1260 |
| South Africa, Free State, Fairview | -27.3794 | 27.1027 | 23/04/2010 | 2 | 370 |
| South Africa, Northern Cape, Vaalkoppies Perseel 203 | -28.4334 | 21.2498 | 23/04/2010 | 2 | 400 |
| South Africa, Northern Cape, Klein Papkuil | -28.4833 | 23.7 | 23/04/2010 | 2 | 70 |
| South Africa, Northern Cape, Dronfield | -28.6166 | 24.8 | 23/04/2010 | 2 | 76 |
| South Africa, Northern Cape, Geluksoord | -28.5474 | 21.7675 | 23/04/2010 | 2 | 350 |
| South Africa, Northern Cape, Vryheids Trust | -29.0274 | 23.7302 | 25/04/2010 | 2 | 68 |
| South Africa, Eastern Cape, De Vrede | -32.3788 | 24.3694 | 26/04/2010 | 2 | 2000 |
| South Africa, Northern Cape, Baklei-Eiland Perseel 219 | -28.6625 | 21.0722 | 27/04/2010 | 2 | 200 |
| South Africa, Northern Cape, Wegdraai | -28.873 | 21.9389 | 27/04/2010 | 2 | 200 |
| South Africa, Northern Cape, Wildebeestkooij | -30.194 | 23.684 | 27/04/2010 | 2 | 55 |
| South Africa, Northern Cape, Langverwacht | -28.7968 | 20.6275 | 28/04/2010 | 2 | 500 |
| South Africa, Free State, Ferreira | -29.2916 | 26.2166 | 28/04/2010 | 2 | 24 |
| South Africa, Eastern Cape, Tandjiesview | -32.4586 | 24.4616 | 28/04/2010 | 2 | 3000 |
| South Africa, Eastern Cape, Roodebloem | -32.1858 | 24.56 | 30/04/2010 | 2 | 740 |
| South Africa, Free State, Doornvlakte | -29.0558 | 26.0586 | 30/04/2010 | 2 | 100 |
| South Africa, Western Cape, Drie Riviere | -33.1858 | 22.04202 | 02/05/2010 | 2 | 733 |
| South Africa, Northern Cape, Irene | -29.2025 | 23.5919 | 02/05/2010 | 2 | 638 |
| South Africa, Western Cape, Swartrivier | -33.1719 | 22.02808 | 03/05/2010 | 2 | 304 |
| South Africa, Northern Cape, Oumurasie | -28.8768 | 22.026 | 03/05/2010 | 2 | 300 |
| South Africa, Northern Cape, Badenhorstfontein | -28.7561 | 24.7638 | 03/05/2010 | 2 | 280 |
| South Africa, Northern Cape, De Hoek | -29.6863 | 23.0463 | 04/05/2010 | 2 | 673 |
| South Africa, North West, Tweebuffels | -26.1333 | 26.5 | 05/05/2010 | 2 | 475 |
| South Africa, Western Cape, Vlagfontein | -32.5061 | 22.0416 | 06/05/2010 | 2 | 1700 |
| South Africa, Eastern Cape, Schoonfontein | -34.0833 | 24.4769 | 06/05/2010 | 2 | 8000 |
| South Africa, Western Cape, Bulwater | -32.5006 | 21.8147 | 06/05/2010 | 2 | 300 |
| South Africa, Western Cape, Melkhout | -34.0022 | 20.22897 | 06/05/2010 | 2 | 400 |
| South Africa, North West, Klipbankfontein | -26.1166 | 26.1666 | 07/05/2010 | 2 | NA |
| South Africa, North West, Doornhoek | -26.15 | 26.3 | 07/05/2010 | 2 | NA |
| South Africa, Western Cape, Pypfontein | -32.0408 | 23.4641 | 07/05/2010 | 2 | 200 |
| Namibia, Hardap, Orion | -25.44 | 17.19 | 10/05/2010 | 2 | 2100 |
| Namibia, Hardap, Driedoring | -24.31 | 18.06 | 10/05/2010 | 2 | 356 |
| Namibia, Hardap, Hebron No 493 | -25.5597 | 19.69 | 10/05/2010 | 2 | 3300 |
| South Africa, Free State, Leeupoort | -29.8111 | 26.195 | 10/05/2010 | 2 | 1200 |
| South Africa, Northern Cape, Geduld | -29.516 | 22.5941 | 11/05/2010 | 2 | NA |
| South Africa, Eastern Cape, Groenakkers | -33.9309 | 24.972 | 11/05/2010 | 2 | 8000 |
| South Africa, Gauteng, Mackay Estates | -26.6418 | 27.957 | 13/05/2010 | 2 | 20 |
| South Africa, Limpopo, Kwaggavlakte | -24.5693 | 27.4409 | 14/05/2010 | 2 | 30 |
| South Africa, North West, De Paarl | -26.1166 | 26 | 14/05/2010 | 2 | 160 |
| Namibia, Hardap, Dassiesfontein 1 | -24.56 | 17.69 | 15/05/2010 | 2 | 45 |
| Namibia, Hardap, Toeloop | -24.69 | 18.19 | 15/05/2010 | 2 | 756 |
| South Africa, Northern Cape, Doornrivier | -28.8484 | 22.02168 | 15/05/2010 | 2 | 130 |
| Namibia, Hardap, Hardap Plots # 24 | -24.44 | 17.81 | 15/05/2010 | 2 | 16 |
| Namibia, Hardap, Donkerhoek | -25.56 | 19.81 | 16/05/2010 | 2 | 250 |
| South Africa, Western Cape, Klipdrift Wcp | -33.8358 | 19.9219 | 17/05/2010 | 2 | 34 |
| South Africa, Western Cape, Ierfontein | -32.1602 | 23.5247 | 17/05/2010 | 2 | NA |
| South Africa, Eastern Cape, Opreisfontein | -32.25 | 24.75 | 20/05/2010 | 2 | 200 |
| Namibia, Hardap, Groot Karris | -24.31 | 17.69 | 22/05/2010 | 2 | 378 |
| South Africa, Eastern Cape, Kromhoud Dairy | -34.1398 | 24.4487 | 25/05/2010 | 2 | NA |
| South Africa, North West, Blaauwwildebeestput | -26.5 | 25.8166 | 26/05/2010 | 2 | NA |
| South Africa, Northern Cape, Mugglestone2 | -31.3957 | 23.1472 | 26/05/2010 | 2 | 360 |
| South Africa, Eastern Cape, Gradita | -34.0163 | 23.9947 | 27/05/2010 | 2 | 6000 |
| South Africa, Western Cape, Leeukraal | -32.6817 | 22.0522 | 27/05/2010 | 2 | NA |
| South Africa, Western Cape, Branddoorns | -32.3909 | 22.2943 | 27/05/2010 | 2 | 300 |
| South Africa, Eastern Cape, Two Streams | -33.9732 | 24.50508 | 27/05/2010 | 2 | 1700 |
| South Africa, Eastern Cape, De Puts | -32.9527 | 22.9821 | 27/05/2010 | 2 | 500 |
| South Africa, Western Cape, Rhenosterkop | -32.7691 | 22.9537 | 28/05/2010 | 2 | 100 |
| South Africa, Eastern Cape, Grasslands Ecp | -33.0016 | 23.9727 | 28/05/2010 | 2 | 7000 |
| South Africa, Western Cape, Werda | -33.8537 | 18.96847 | 29/05/2010 | 2 | 485 |
| Botswana, South-East, Seribe | -24.8133 | 25.8303 | 30/05/2010 | 2 | 3122 |
| South Africa, Northern Cape, Cypherkuil | -30.5716 | 24.6553 | 31/05/2010 | 2 | NA |
| Namibia, Karas, Ramansdrift 135 | -28.8 | 18.3 | 03/06/2010 | 2 | 139 |
| South Africa, Western Cape, Hoeko | -34.0158 | 20.2188 | 03/06/2010 | 2 | 420 |
| Namibia, Erongo, Eitavere 150 | -21.19 | 15.19 | 07/06/2010 | 2 | 300 |
| South Africa, Western Cape, Spesbona | -34.0764 | 19.3765 | 08/06/2010 | 2 | 450 |
| South Africa, Western Cape, Mierfontein | -34.0632 | 20.9017 | 09/06/2010 | 2 | 1500 |
| Namibia, Karas, Grasswater 150 | -28.56 | 19.31 | 09/06/2010 | 2 | 600 |
| South Africa, Northern Cape, Louisvale Weg | -28.5333 | 21.2994 | 10/06/2010 | 2 | 600 |
| South Africa, Western Cape, Avondrust | -34.1065 | 18.3682 | 13/06/2010 | 2 | 30 |
| Namibia, Hardap, Brynard | -24.06 | 19.19 | 14/06/2010 | 2 | 1 |
| South Africa, Western Cape, Welgeluk | -33.642 | 22.1791 | 15/06/2010 | 2 | 173 |
| South Africa, Free State, Goedverwacht | -27.1969 | 28.6666 | 19/06/2010 | 2 | 1600 |
| South Africa, Free State, Fisher | -27.2616 | 27.5553 | 21/06/2010 | 2 | 191 |
| South Africa, Western Cape, Mw Boerdery | -34.0182 | 20.9405 | 22/06/2010 | 2 | 900 |
| South Africa, Western Cape, Vergelegen | -34.0801 | 18.8941 | 20/07/2010 | 3 | 202 |
| South Africa, Northern Cape, Oranjezucht | -29.5169 | 22.4424 | 18/08/2010 | 3 | 850 |
| Mozambique, Zambezia, Nzanzia | -17.8329 | 36.0626 | 01/09/2010 | 3 | NA |
| Mozambique, Zambezia, Deda | -17.9916 | 35.7405 | 01/09/2010 | 3 | NA |
| Mozambique, Zambezia, Nuere | -17.8329 | 36.0626 | 01/09/2010 | 3 | NA |
| Mozambique, Zambezia, Chimuara Mopeia Zambezia Mozambique | -17.7833 | 35.4028 | 01/09/2010 | 3 | NA |
| Mauritania, Adrar, Village Of Meddah | 19.19 | -13.41 | 25/10/2010 | 4 | 300 |
| Mauritania, Adrar, Village Of Tawaz | 20.31 | -13.03 | 10/11/2010 | 4 | 120 |
| Mauritania, Inchiri, Village Of Akjoujt | 20.45 | -14.23 | 11/11/2010 | 4 | 370 |
| South Africa, Western Cape, Farm 137 | -34.055 | 22.2052 | 24/12/2010 | 4 | 45 |
| Mozambique, Maputo, Boane | -26.0347 | 32.35269 | 01/01/2011 | 1 | 28 |
| Mozambique, Maputo, Magude | -24.7018 | 32.44014 | 01/01/2011 | 1 | 34 |
| Mozambique, Maputo, Moamba | -25.3204 | 32.32315 | 01/01/2011 | 1 | 64 |
| Mozambique, Maputo, Matutine | -26.5272 | 32.56355 | 01/01/2011 | 1 | 131 |
| Mozambique, Maputo, Manhica Maputo Mozambique | -25.2023 | 32.81005 | 01/01/2011 | 1 | 65 |
| Mozambique, Maputo, Marracuene | -25.6572 | 32.71213 | 01/01/2011 | 1 | 82 |
| South Africa, Western Cape, Drakenstein | -33.6491 | 18.9605 | 02/01/2011 | 1 | 71 |
| South Africa, Eastern Cape, Rvf2011_Ecp_007 | -32.6789 | 28.24389 | 10/01/2011 | 1 | 1500 |
| South Africa, Eastern Cape, Rvf2011_Ecp_011 | -32.2 | 24.4 | 24/01/2011 | 1 | 400 |
| South Africa, Eastern Cape, Graaf-Reinet | -32.3113 | 24.4597 | 26/01/2011 | 1 | 50 |
| South Africa, Eastern Cape, Rvf2011_Ecp_006 | -32.3944 | 24.43722 | 28/01/2011 | 1 | 1200 |
| South Africa, Eastern Cape, Rvf_Ecp_053 | -32.3811 | 25.01111 | 03/02/2011 | 1 | 490 |
| South Africa, Eastern Cape, Rvf2011_Ecp_012 | -32.7683 | 28.01167 | 04/02/2011 | 1 | 337 |
| South Africa, Eastern Cape, Rvf2011_Ecp_009 | -32.0667 | 24.36667 | 10/02/2011 | 1 | 100 |
| South Africa, Eastern Cape, Rvf2011_Ecp_021 | -32.55 | 24.1 | 10/02/2011 | 1 | 5000 |
| South Africa, Eastern Cape, Rvf2011_Ecp_010 | -32.2833 | 24.2333 | 10/02/2011 | 1 | 2000 |
| South Africa, Eastern Cape, Rvf2011_Ecp_020 | -32.2833 | 24.26667 | 10/02/2011 | 1 | 1000 |
| South Africa, Eastern Cape, Rvf_Ecp_029 | -32.1667 | 24.3 | 10/02/2011 | 1 | 2000 |
| South Africa, Eastern Cape, Rvf2011_Ecp_004 | -32.1741 | 24.36836 | 15/02/2011 | 1 | 16 |
| South Africa, Eastern Cape, Rvf2011_Ecp_026 | -32.3422 | 24.38078 | 15/02/2011 | 1 | 3000 |
| South Africa, Eastern Cape, Rvf2011_Ecp_025 | -32.0778 | 24.67833 | 16/02/2011 | 1 | 1 |
| South Africa, Eastern Cape, Rvf2011_Ecp_003 | -32.4314 | 24.66667 | 17/02/2011 | 1 | 3000 |
| South Africa, Eastern Cape, Rvf2011_Ecp_005 | -32.7273 | 24.47758 | 17/02/2011 | 1 | 3000 |
| South Africa, Eastern Cape, Rvf2011_Ecp_002 | -32.1322 | 24.44081 | 20/02/2011 | 1 | 16 |
| South Africa, Northern Cape, Rvf2011_Ncp_001 | -31.2833 | 24.29892 | 25/02/2011 | 1 | 1000 |
| South Africa, Western Cape, Rvf2011_Wcp_003 | -32.4642 | 22.98528 | 01/03/2011 | 1 | 140 |
| South Africa, Northern Cape, Rfv_Ncp_013 | -31.4414 | 23.88969 | 02/03/2011 | 1 | 1960 |
| South Africa, Western Cape, Rvf2011_Wcp_13 | -32.3914 | 23.02361 | 02/03/2011 | 1 | 4500 |
| South Africa, Eastern Cape, Rvf2011_Ecp_015 | -31.8965 | 24.7795 | 03/03/2011 | 1 | 150 |
| South Africa, Eastern Cape, Rvf2011_Ecp_019 | -32.0089 | 24.42889 | 07/03/2011 | 1 | 2 |
| South Africa, Western Cape, Rvf2011_Wcp_006 | -34.0175 | 23.36861 | 07/03/2011 | 1 | 40 |
| South Africa, Eastern Cape, Rvf2011_Ecp_008 | -33.1667 | 24.6667 | 07/03/2011 | 1 | 6 |
| South Africa, Western Cape, Rvf_Wcp_022 | -32.5183 | 22.36139 | 07/03/2011 | 1 | 300 |
| South Africa, Western Cape, Rvf2011_Wcp_005 | -32.1144 | 23.00222 | 07/03/2011 | 1 | 416 |
| South Africa, Eastern Cape, Rvf_Ecp_062 | -31.3567 | 25.01737 | 10/03/2011 | 1 | 250 |
| South Africa, Eastern Cape, Rvf_Ecp_061 | -31.668 | 24.73153 | 10/03/2011 | 1 | 300 |
| South Africa, Western Cape, Rvf2011_Wcp_010 | -32.4242 | 22.84167 | 10/03/2011 | 1 | 1100 |
| South Africa, Eastern Cape, Rvf2011_Ecp_023 | -32.5931 | 23.99767 | 10/03/2011 | 1 | 40 |
| South Africa, Western Cape, Rvf2011_Wcp_011 | -33.774 | 19.52139 | 10/03/2011 | 1 | 165 |
| South Africa, Western Cape, Rvf2011_Wcp_009 | -32.3764 | 22.59556 | 10/03/2011 | 1 | 94 |
| South Africa, Western Cape, Rvf2011_Wcp_004 | -31.9599 | 23.00222 | 10/03/2011 | 1 | 1200 |
| South Africa, Western Cape, Rvf2011_Wcp_007 | -31.9167 | 23.03333 | 11/03/2011 | 1 | 80 |
| South Africa, Western Cape, Rvf2011_Wcp_008 | -32.4006 | 22.42333 | 14/03/2011 | 1 | 4 |
| South Africa, Eastern Cape, Rvf2011_Ecp_022 | -32.1637 | 24.24183 | 15/03/2011 | 1 | 1000 |
| South Africa, Eastern Cape, Rvf2011_Ecp_024 | -32.5146 | 24.476 | 15/03/2011 | 1 | 400 |
| South Africa, Western Cape, Rvf2011_Wcp_006 | -32.5981 | 23.07667 | 15/03/2011 | 1 | 22 |
| South Africa, Western Cape, Rvf2011_Wcp_14 | -32.235 | 23.6425 | 15/03/2011 | 1 | 200 |
| South Africa, Eastern Cape, Rvf2011_Ecp_014 | -32.3667 | 23.78333 | 16/03/2011 | 1 | 100 |
| South Africa, Western Cape, Rvf2011_Wcp_15 | -31.7994 | 23.73694 | 16/03/2011 | 1 | 200 |
| South Africa, Eastern Cape, Rvf2011_Ecp_013 | -32.6486 | 24.03778 | 16/03/2011 | 1 | 25 |
| South Africa, Northern Cape, Rvf_Ncp_009 | -30.6946 | 24.00819 | 18/03/2011 | 1 | 16 |
| South Africa, Eastern Cape, Rvf_Ecp_060 | -31.3858 | 25.14722 | 18/03/2011 | 1 | 250 |
| South Africa, Western Cape, Rvf2011_Wcp_012 | -33.0773 | 20.595 | 18/03/2011 | 1 | 140 |
| South Africa, Eastern Cape, Rvf2011_Ecp_018 | -31.8122 | 24.80083 | 18/03/2011 | 1 | 100 |
| South Africa, Western Cape, Rvf2011_Wcp_12 | -32.2686 | 22.74278 | 18/03/2011 | 1 | 500 |
| South Africa, Northern Cape, Rvf_Ncp_002 | -31.0346 | 24.83789 | 20/03/2011 | 1 | 800 |
| South Africa, Eastern Cape, Rvf_Ecp_073 | -31.6395 | 24.64275 | 22/03/2011 | 1 | 150 |
| South Africa, Eastern Cape, Rvf_Ecp_070 | -32.0166 | 25.31347 | 22/03/2011 | 1 | 650 |
| South Africa, Eastern Cape, Rvf_Ecp_042 | -33.0333 | 24.88333 | 22/03/2011 | 1 | 300 |
| South Africa, Eastern Cape, Rvf_Ecp_048 | -32.3667 | 23.6 | 22/03/2011 | 1 | 2000 |
| South Africa, Western Cape, Rvf2011_Wcp_17 | -32.9788 | 22.94978 | 24/03/2011 | 1 | 200 |
| South Africa, Northern Cape, Rvf_Ncp_003 | -30.6887 | 25.3528 | 25/03/2011 | 1 | 480 |
| South Africa, Eastern Cape, Rvf2011_Ecp_027 | -33.3333 | 24.76667 | 25/03/2011 | 1 | 300 |
| South Africa, Eastern Cape, Rvf2011_Ecp_017 | -32.4722 | 24.6675 | 25/03/2011 | 1 | 1500 |
| South Africa, Eastern Cape, Rvf_Ecp_041 | -32.5167 | 25.25 | 26/03/2011 | 1 | 300 |
| South Africa, Eastern Cape, Rvf_Ecp_052 | -32.9008 | 25.91 | 28/03/2011 | 1 | 1500 |
| South Africa, Eastern Cape, Rvf_Ecp_039 | -32.8 | 25.775 | 28/03/2011 | 1 | 1400 |
| South Africa, Eastern Cape, Rvf_Ecp_051 | -32.8778 | 25.64444 | 28/03/2011 | 1 | 1500 |
| South Africa, Western Cape, Rvf_Wcp_024 | -31.9047 | 23.45897 | 29/03/2011 | 1 | 22 |
| South Africa, Western Cape, Rvf2011_Wcp_16 | -32.0714 | 23.86333 | 29/03/2011 | 1 | 4 |
| South Africa, Eastern Cape, Rvf_Ecp_043 | -32.8333 | 25.625 | 29/03/2011 | 1 | 1150 |
| South Africa, Western Cape, Rvf_Wcp_023 | -32.2 | 23.18333 | 30/03/2011 | 1 | 85 |
| South Africa, Eastern Cape, Rvf_Ecp_044 | -32.25 | 24.16667 | 31/03/2011 | 1 | 1 |
| South Africa, Eastern Cape, Rvf_Ecp_045 | -32.0333 | 24.21667 | 31/03/2011 | 1 | 2000 |
| South Africa, Western Cape, Rvf2011_Wcp_19 | -33.2636 | 21.87861 | 31/03/2011 | 1 | 183 |
| South Africa, Eastern Cape, Rvf2011_Ecp_016 | -32.8036 | 24.47417 | 31/03/2011 | 1 | 10000 |
| South Africa, Western Cape, Rvf2011_Wcp_20 | -32.4006 | 22.4233 | 01/04/2011 | 2 | 412 |
| South Africa, Northern Cape, Rvf_Ncp_012 | -31.8167 | 22.13333 | 01/04/2011 | 2 | 200 |
| South Africa, Northern Cape, Rvf_Ncp_007 | -30.4108 | 20.94472 | 01/04/2011 | 2 | 550 |
| South Africa, Western Cape, Rvf_Wcp_025 | -34.0428 | 22.16094 | 02/04/2011 | 2 | 374 |
| South Africa, Northern Cape, Rvf_Ncp_011 | -30.4888 | 19.94219 | 04/04/2011 | 2 | 500 |
| Namibia, Oshikoto, Omuthiya | -18.35 | 16.58 | 04/04/2011 | 2 | 40 |
| South Africa, Western Cape, Rvf_Wcp_032 | -32.9086 | 19.30083 | 04/04/2011 | 2 | 380 |
| South Africa, Eastern Cape, Rvf_Ecp_054 | -32.6278 | 25.95583 | 04/04/2011 | 2 | 300 |
| South Africa, Eastern Cape, Rvf_Ecp_066 | -31.8877 | 25.48797 | 04/04/2011 | 2 | 627 |
| South Africa, Eastern Cape, Rvf_Ecp_050 | -32.1667 | 24.8 | 05/04/2011 | 2 | 200 |
| South Africa, Western Cape, Rvf_Wcp_033 | -33.5 | 18.79194 | 05/04/2011 | 2 | 400 |
| South Africa, Eastern Cape, Rvf_Ecp_031 | -32.1859 | 24.56003 | 05/04/2011 | 2 | 2 |
| South Africa, Western Cape, Rvf2011_Wcp_18 | -33.1733 | 22.03083 | 05/04/2011 | 2 | 4 |
| South Africa, Eastern Cape, Rvf_Ecp_033 | -32.05 | 24.8 | 05/04/2011 | 2 | 200 |
| South Africa, Eastern Cape, Rvf_Ecp_040 | -32.4054 | 24.07583 | 05/04/2011 | 2 | 400 |
| South Africa, Eastern Cape, Rfv_Ecp_072 | -32.4481 | 23.36333 | 05/04/2011 | 2 | 270 |
| South Africa, Western Cape, Rvf_Wcp_042 | -32.5061 | 22.04167 | 08/04/2011 | 2 | 1 |
| South Africa, Western Cape, Rvf_Wcp_028 | -32.7725 | 22.48694 | 08/04/2011 | 2 | 297 |
| South Africa, Eastern Cape, Rvf2011_Ecp_28 | -32.1167 | 24.35 | 08/04/2011 | 2 | 250 |
| South Africa, Northern Cape, Rfv_Ncp_010 | -31.2492 | 21.15917 | 08/04/2011 | 2 | 250 |
| South Africa, Western Cape, Rvf_Wcp_041 | -32.5061 | 22.04167 | 08/04/2011 | 2 | 895 |
| South Africa, Eastern Cape, Rvf_Ecp_047 | -32.6833 | 24.64278 | 08/04/2011 | 2 | 1000 |
| South Africa, Eastern Cape, Rvf_Ecp_049 | -32.0061 | 24.80139 | 08/04/2011 | 2 | 2000 |
| South Africa, Eastern Cape, Rvf_Ecp_032 | -33.15 | 23.75 | 08/04/2011 | 2 | 1000 |
| South Africa, Eastern Cape, Rvf_Ecp_064 | -32.0326 | 25.54189 | 09/04/2011 | 2 | 730 |
| South Africa, Eastern Cape, Rvf_Ecp_046 | -33.0203 | 25.78667 | 09/04/2011 | 2 | 800 |
| South Africa, Western Cape, Rvf_Wcp_027 | -32.3783 | 22.81222 | 09/04/2011 | 2 | 2500 |
| South Africa, Eastern Cape, Rvf_Ecp_074 | -33.33 | 24.88186 | 10/04/2011 | 2 | 235 |
| South Africa, Western Cape, Rvf_Wcp_030 | -32.9086 | 18.80889 | 10/04/2011 | 2 | 1100 |
| South Africa, Eastern Cape, Rvf_Ecp_034 | -32.3833 | 24.05 | 11/04/2011 | 2 | 150 |
| South Africa, Eastern Cape, Rvf_Ecp_035 | -31.8884 | 26.86925 | 11/04/2011 | 2 | 200 |
| South Africa, Eastern Cape, Rvf_Ecp_036 | -33.0478 | 24.33083 | 11/04/2011 | 2 | 200 |
| South Africa, Eastern Cape, Rvf_Ecp_037 | -32.2963 | 24.00206 | 11/04/2011 | 2 | 200 |
| South Africa, Northern Cape, Rvf_Ncp_004 | -29.8021 | 24.4005 | 11/04/2011 | 2 | 300 |
| South Africa, Eastern Cape, Rvf_Ecp_068 | -31.5574 | 24.93083 | 13/04/2011 | 2 | 100 |
| South Africa, Eastern Cape, Rvf_Ecp_063 | -31.903 | 25.45386 | 13/04/2011 | 2 | 840 |
| South Africa, Eastern Cape, Rvf_Ecp_065 | -31.3849 | 25.07617 | 14/04/2011 | 2 | 10 |
| South Africa, Western Cape, Rvf_Wcp_026 | -32.8717 | 23.06361 | 14/04/2011 | 2 | 1520 |
| South Africa, Eastern Cape, Rvf_Ecp_055 | -34.0017 | 23.93944 | 15/04/2011 | 2 | 6000 |
| South Africa, Eastern Cape, Rvf_Ecp_069 | -31.555 | 24.80631 | 15/04/2011 | 2 | 305 |
| South Africa, Western Cape, Rvf_Wcp_040 | -32.6628 | 22.93028 | 15/04/2011 | 2 | 2400 |
| South Africa, Northern Cape, Rvf_Ncp_006 | -31.2587 | 22.28208 | 15/04/2011 | 2 | 1509 |
| South Africa, Eastern Cape, Rvf_Ecp_038 | -32.8611 | 25.55278 | 18/04/2011 | 2 | 1 |
| South Africa, Eastern Cape, Rvf_Ecp_030 | -32.4 | 23.71667 | 18/04/2011 | 2 | 800 |
| South Africa, Eastern Cape, Rvf_Wcp_051 | -33.2167 | 22.86667 | 19/04/2011 | 2 | 600 |
| South Africa, Northern Cape, Rvf_Ncp_005 | -30.8049 | 24.41778 | 20/04/2011 | 2 | 35 |
| South Africa, Western Cape, Rvf_Wcp_029 | -32.8215 | 19.26311 | 23/04/2011 | 2 | 1100 |
| South Africa, Western Cape, Rvf_Wcp_043 | -34.4139 | 19.42083 | 28/04/2011 | 2 | 500 |
| South Africa, Western Cape, Rvf_Wcp_037 | -33.695 | 19.69861 | 04/05/2011 | 2 | 56 |
| South Africa, Eastern Cape, Rvf_Ecp_059 | -34.0458 | 24.16 | 04/05/2011 | 2 | 1600 |
| Namibia, Oshikoto, Omuthiya | -18.35 | 16.58 | 04/05/2011 | 2 | 190 |
| South Africa, Eastern Cape, Rvf_Ecp_067 | -32.1636 | 25.97789 | 09/05/2011 | 2 | 730 |
| South Africa, Western Cape, Rvf_Wcp_031 | -31.8849 | 23.71494 | 11/05/2011 | 2 | 230 |
| South Africa, Eastern Cape, Rvf_Ecp_057 | -34.1358 | 24.59806 | 13/05/2011 | 2 | 1900 |
| South Africa, Western Cape, Rvf_Wcp_036 | -33.0597 | 22.5075 | 13/05/2011 | 2 | 1300 |
| South Africa, Eastern Cape, Rvf_Ecp_058 | -33.9403 | 24.98583 | 20/05/2011 | 2 | 805 |
| South Africa, Eastern Cape, Rvf_Ecp_056 | -34.0044 | 24.21058 | 20/05/2011 | 2 | 105 |
| South Africa, Eastern Cape, Rvf_Ecp_071 | -32.4667 | 23.86667 | 20/05/2011 | 2 | 1000 |
| South Africa, Eastern Cape, Rvf_Ecp_075 | -32.0457 | 25.55031 | 23/05/2011 | 2 | 1300 |
| South Africa, Western Cape, Rvf_Wcp_035 | -33.9083 | 19.10917 | 23/05/2011 | 2 | 15 |
| South Africa, Western Cape, Rvf_Wcp_034 | -33.9675 | 20.01861 | 25/05/2011 | 2 | 430 |
| South Africa, Western Cape, Rvf_Wcp_039 | -31.5867 | 18.2425 | 26/05/2011 | 2 | 20 |
| Namibia, Oshikoto, Onankali | -19.2485 | 17.72203 | 03/06/2011 | 2 | 126 |
| South Africa, Northern Cape, Rvf_Ncp_008 | -28.4503 | 21.305 | 06/06/2011 | 2 | 300 |
| South Africa, Western Cape, Rvf_Wcp_038 | -32.9354 | 18.76325 | 09/06/2011 | 2 | 3230 |
| South Africa, Western Cape, Rvf_Wcp_044 | -34.0651 | 18.87178 | 23/07/2011 | 3 | 12 |
| South Africa, Western Cape, Rvf_Wcp_045 | -32.4006 | 22.42333 | 29/07/2011 | 3 | NA |
| Comoros, Anjouan, Anjouan Anjouan Comoros | -12.25 | 44.41667 | 01/08/2011 | 3 | 53 |
| Comoros, Ngazidja, Moheli | -11.6518 | 43.33092 | 01/08/2011 | 3 | 48 |
| Comoros, Ngazidja, Grande Comore Ngazidja Comoros | -11.5861 | 43.33306 | 01/08/2011 | 3 | 174 |
| Mauritania, Trarza, Ouad Naga | 18.42169 | -15.4062 | 15/09/2012 | 3 | NA |
| Mauritania, Hodh Ech Chargi, Amourj | 15.89625 | -6.94514 | 16/09/2012 | 3 | NA |
| Mauritania, Hodh El Gharbi, Tintane | 15.95279 | -10.2937 | 16/09/2012 | 3 | NA |
| Mauritania, Hodh Ech Chargi, Djigueni | 16.01854 | -8.78456 | 16/09/2012 | 3 | NA |
| Mauritania, Brakna, Bababe | 16.4566 | -13.9482 | 16/09/2012 | 3 | NA |
| Mauritania, Hodh Ech Chargi, Nema | 16.45943 | -7.32705 | 16/09/2012 | 3 | NA |
| Mauritania, Trarza, Rosso | 16.65718 | -15.6942 | 16/09/2012 | 3 | NA |
| Mauritania, Assaba, Kiffa | 16.69059 | -11.3425 | 16/09/2012 | 3 | NA |
| Mauritania, Assaba, Guerou | 16.87571 | -11.9299 | 16/09/2012 | 3 | NA |
| Mauritania, Brakna, Aleg | 16.99554 | -13.6803 | 16/09/2012 | 3 | NA |
| Mauritania, Hodh El Gharbi, Tamchekket | 17.11696 | -10.4434 | 16/09/2012 | 3 | NA |
| Mauritania, Brakna, Magta-Lahjar | 17.65013 | -13.0614 | 16/09/2012 | 3 | NA |
| Mauritania, Tagant, Moudjeria | 18.01249 | -12.1619 | 16/09/2012 | 3 | NA |
| Mauritania, Tagant, Tidjikja | 18.44075 | -11.5504 | 16/09/2012 | 3 | NA |
| Mauritania, Hodh Ech Chargi, Nbeiket Lahouch | 19.33921 | -7.00146 | 16/09/2012 | 3 | NA |
| Senegal, Kedougou, Baya Village Kedougoutambacounda Senegal | 12.46388 | -12.4683 | 16/10/2012 | 4 | NA |
| Kenya, North Eastern, Ijara District Kenya | -1.58111 | 40.52028 | 01/05/2013 | 2 | 1396 |
| Senegal, Saint Louis, Gueumbeul | 15.8993 | -16.5138 | 10/09/2013 | 3 | 33 |
| Senegal, Saint Louis, Diama Peulh Diama Ndiaye | 15.97121 | -16.3228 | 16/09/2013 | 3 | 168 |
| Mauritania, Trarza, Chgara | 16.65718 | -15.6942 | 17/09/2013 | 3 | 150 |
| Senegal, Saint Louis, Guinthe Ndiaye Ngnith Ndiaye | 15.97121 | -16.3228 | 19/09/2013 | 3 | 40 |
| Mauritania, Trarza, Pk 60 | 17.11608 | -15.7724 | 01/10/2013 | 4 | 100 |
| Mauritania, Brakna, Tadressa | 17.65013 | -13.0614 | 08/10/2013 | 4 | 300 |
| Senegal, Dakar, Sangalkhame | 14.82679 | -17.2457 | 10/10/2013 | 4 | 190 |
| Mauritania, Brakna, Kadjel Abou | 16.4566 | -13.9482 | 10/10/2013 | 4 | 350 |
| Mauritania, Guidimakha, Selibaby | 15.1889 | -12.3074 | 27/10/2013 | 4 | 250 |
| Chad, Lac, Lac Bol Chad | 13.75051 | 14.17699 | 01/01/2014 | 1 | 924 |
| Botswana, Chobe, Mabele Tinto Crush Chobe West | -18.3967 | 24.71014 | 25/07/2014 | 3 | 25 |
| Botswana, South-East, Bela Bela Gaborone | -24.6753 | 25.9729 | 07/08/2014 | 3 | 55 |
| Mauritania, Brakna, Brakna | 17.20923 | -13.7513 | 29/09/2015 | 3 | 800 |
| Mauritania, Tagant, Moudjaria | 17.848 | -12.254 | 06/10/2015 | 4 | 72 |
| Mauritania, Brakna, Magta Lahjar Ville | 17.8 | -12.989 | 09/10/2015 | 4 | 450 |
| Mauritania, Assaba, Kiffa | 16.625 | -11.405 | 11/10/2015 | 4 | 700 |
| Uganda, Kabale, Kamuganguzi | -1.26595 | 29.99283 | 03/03/2016 | 1 | NA |
| Uganda, Kabale, Kabale | -1.26595 | 29.99283 | 04/03/2016 | 1 | NA |
| Uganda, Kabale, Kashenyi | -1.24196 | 29.98562 | 15/03/2016 | 1 | 247939 |

**Table S2.** Details of all environmental and habitat geo-spatial variables used for modelling. For analysis, all variables were reduced in latitudinal extent to 37^o^N 40.5^o^S and longitudinal extent to 54^o^E 18^o^W so as to cover the African continent and resampled to a 0.0416^o^ grid cell size using a World Geodetic System 84 projection using ‘raster’ ([Hijmans & van Etten 2012](#_ENREF_9)). Source 1 represents ([Hijmans *et al.* 2005](#_ENREF_8)); 2 ([Brakenridge 2010](#_ENREF_1)); 3 ([Robinson *et al.* 2014](#_ENREF_10)); 4 ([FAO Empres-i database 2016](#_ENREF_4)); 5 ([Chini, Hurtt & Frolking 2014](#_ENREF_2)); 6 ([HarvestChoice 2014](#_ENREF_6)); 7 ([CIESIN 2005](#_ENREF_3)); 8 ([Friedl *et al.* 2010](#_ENREF_5)); 9 ([Hengl *et al.* 2015](#_ENREF_7)).

| **Variable Description** | **Original Spatial Extent** | **Original Spatial Resolution (cell size at equator)** | **Temporal Resolution** | **Source** |
| --- | --- | --- | --- | --- |
| Bioclim – 19 climatic variables | Global | 1km | 2012 | Worldclim (1) |
| Floods and high rainfall events | Africa | Points | 1998-2009 | Dartmouth Flood Observatory (2) |
| Gridded Livestock of the World | Global | 5.6km | 2007 | Gridded Livestock of the World (3) |
| Animals at risk | Africa | Points | 2004-2016 | EMPRES-i (4) |
| Cultivation coverage | Global | 55km | 2010 | Harmonized Land Cover (5) |
| Irrigation | Africa | 10km | 2014 | HarvestChoice (6) |
| Human Population | Global | 111km | 2010 | Gridded Population of the World v3 (7) [(CIESIN 200](#_ENREF_3)5) |
| MODIS Land Cover | Global | 500m | 2012 | MODIS (8) |
| Soil & vegetation characteristics | Africa | 500m | 2012 | World Soil Database (9) |

**Figure S1.** Posterior distributions from an INLA Bayesian additive regression for all regression slopes estimates for (a) Intercept; (b) Annual Precipitation; (c) Annual Precipitation^2^; (d) Annual Mean Temperature; (e) Annual Mean Temperature2; (f) No. of Observed Floods; (g) Sheep Density; (h) Cattle Density; (i) Proportion of Cultivated Land; (j) Proportion of Cultivated Land^2^; (k) Presence of Irrigation; (l) Human Population Density; (m) Land Cover; and (n) Number of Animals at Risk. Labels on x-axis represent slope values and y-axis frequency of value in the model.

**
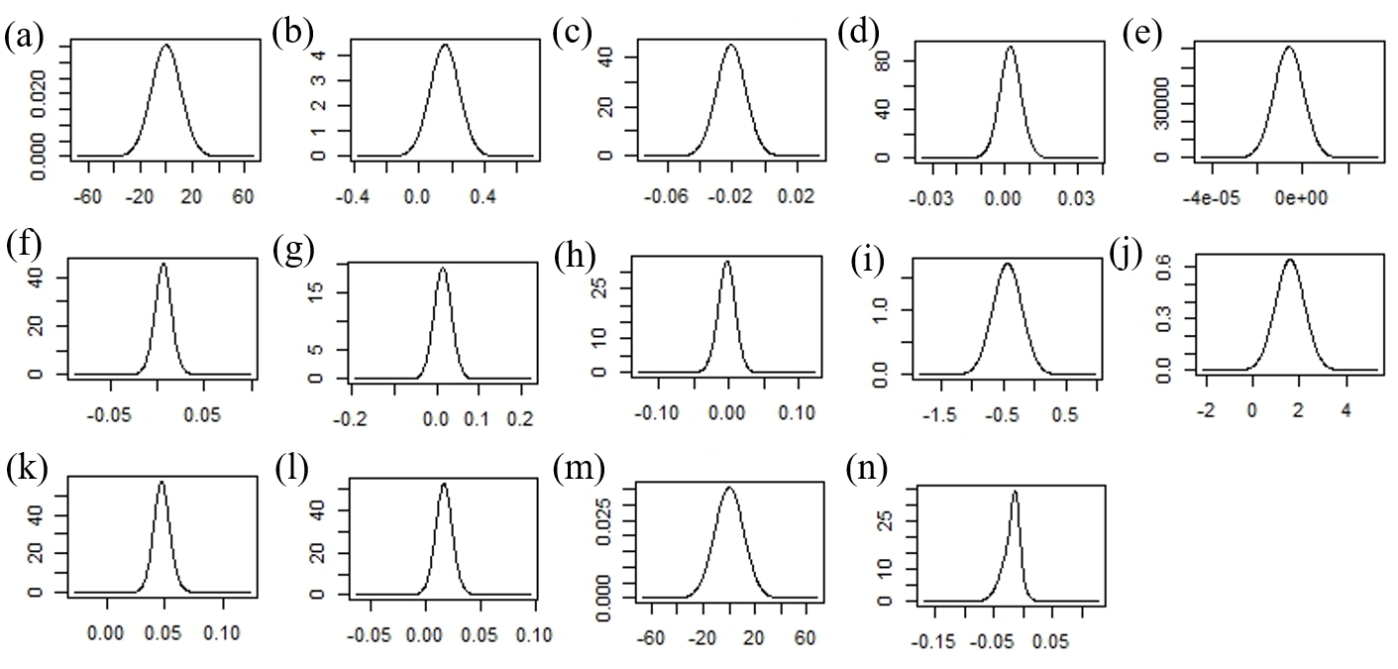
**

**Figure S2.** Histogram of Conditional Predictive Ordinate (CPO) values from a leave-one-out cross validation of all 976 data points in the INLA Bayesian model.


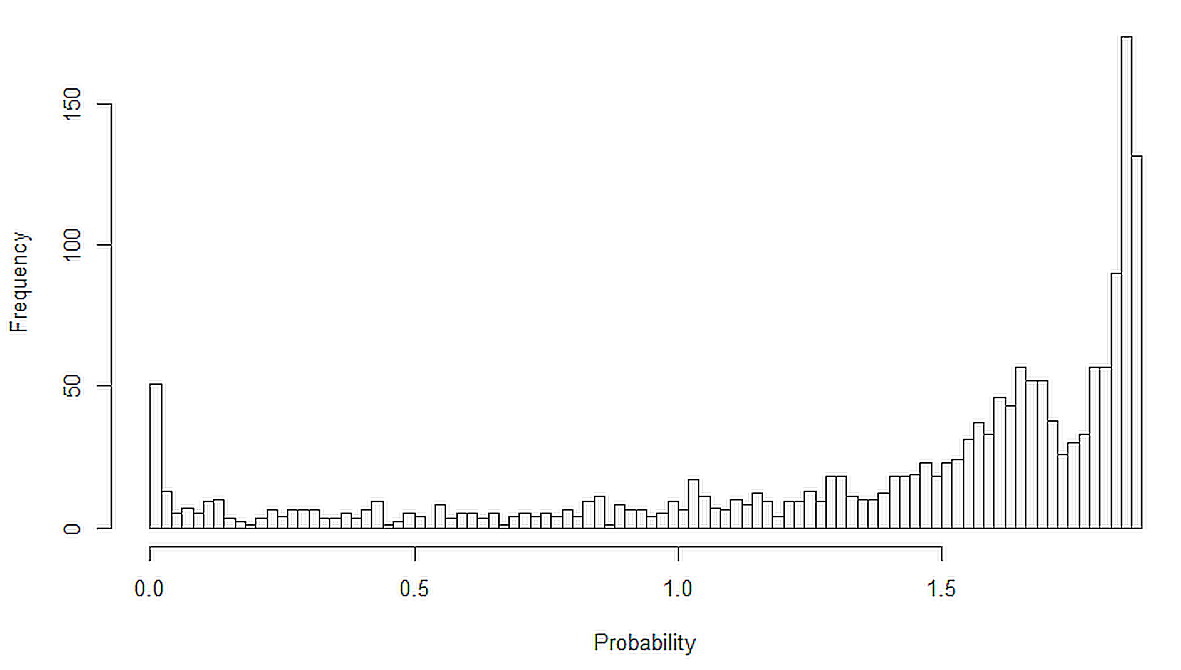


**
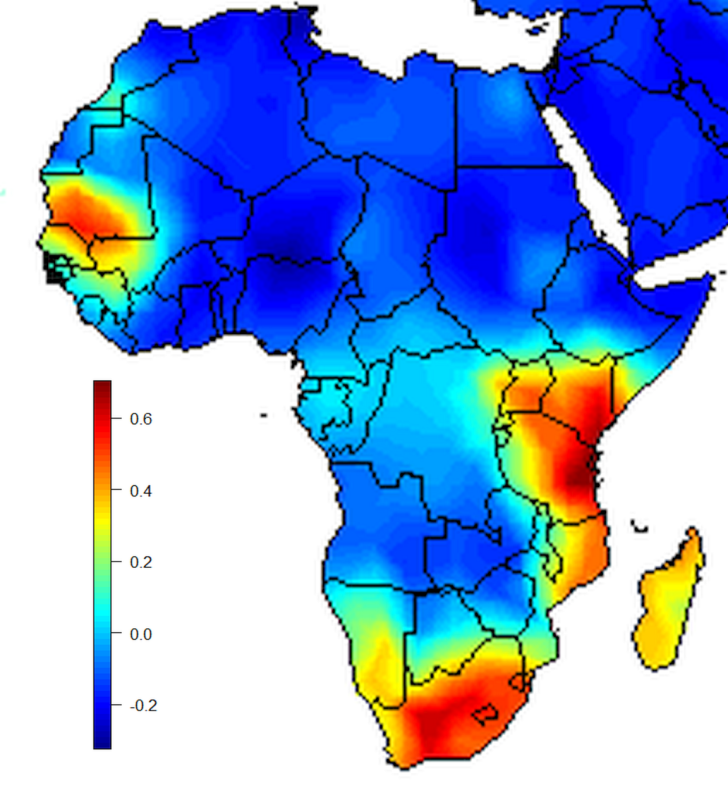
**

**Figure S3.** Plot of the mean component of the Gaussian random field of the final INLA model. Redder areas are locations that have higher data availability and consequently higher predictive confidence for parameters, whereas bluer areas have fewer datapoints and large variance associated with any parameter estimates at those locations.

**SI References**

Brakenridge, G.R. (2010) Global Active Archive of Large Flood Events. (ed. D.F. Observatory). University of Colorado.

Chini, L.P., Hurtt, G.C. & Frolking, S. (2014) Harmonized Global Land Use for Years 1500 – 2100, V1. Data set. Oak Ridge National Laboratory Distributed Active Archive Center, Oak Ridge, Tennessee, .

CIESIN (2005) Gridded Population of the World Version 3 (GPWv3): Population Grids. SEDAC, Columbia University, New York.

FAO Empres-i database (2016) EMPRES Global Animal Disease Information System.

Friedl, M.A., Sulla-Menashe, D., Tan, B., Schneider, A., Ramankutty, N., Sibley, A. & Huang, X. (2010) MODIS Collection 5 global land cover: Algorithm refinements and characterisation of new datasets. *Remote Sensing of the Environment,* **114,** 168-182.

HarvestChoice (2014) Crop Production: SPAM. International Food Policy Research Institute, Washington, DC., and University of Minnesota, St. Paul, MN.

Hengl, T., Heuvelink, G.B.M., Kempen, B., Leenaars, J.G.B., Walsh, M.G., Shepherd, K.D., Sila, A., MacMillan, R.A., Mendes de Jesus, J., Tamene, L. & Tondoh, J.E. (2015) Mapping Soil Properties of Africa at 250 m Resolution: Random Forests Significantly Improve Current Predictions. *PloS one,* **10,** e0125814.

Hijmans, R.J., Cameron, S.E., Parra, J.L., Jones, P.G. & Jarvis, A. (2005) Very high resolution interpolated climate surfaces for global land areas. *International Journal of Climatology,* **25,** 1965-1978.

Hijmans, R.J. & van Etten, J. (2012) Raster: Geographic analysis and modeling with raster data R package

Robinson, T.P., Wint, G.R.W., Conchedda, G., Van Boeckel, T.P., Ercoli, V., Palamara, E., Cinardi, G., D'Aietti, L., Hay, S.I. & Gilbert, M. (2014) Mapping the Global Distribution of Livestock. *PloS one,* **9,** e96084.
